# Distinct modes of RNA degradation by the structurally related coronavirus and arenavirus exoribonucleases

**DOI:** 10.1128/jvi.00798-26

**Published:** 2026-07-23

**Authors:** Patricia C. Hernandez, Nicholas H. Moeller, Hideki Aihara

**Affiliations:** 1 Department of Biochemistry, Molecular Biology and Biophysics, University of Minnesota 200766, Minneapolis, Minnesota, USA; 2 Institute for Molecular Virology, University of Minnesota, Minneapolis, Minnesota, USA; 3 Masonic Cancer Center, University of Minnesota 5635, Minneapolis, Minnesota, USA; The University of Texas Southwestern Medical Center, Dallas, Texas, USA

**Keywords:** viral RNA, antiviral immunity, processivity, Lassa virus, SARS-CoV-2, exoribonuclease

## Abstract

Coronaviruses and arenaviruses encode evolutionarily related DEDDh-family 3′–5′ exoribonuclease (ExoN) domains, which support the replication of their RNA genomes and evasion of the host’s antiviral immunity. Here, we report comparative biochemical analyses of SARS-CoV-2 and Lassa virus (LASV) ExoN, which show that the two enzymes have distinct characteristics despite structural conservation and their shared preference for double-stranded RNA (dsRNA) substrates. SARS-CoV-2 nsp14/10 exhibits a highly processive behavior and tends to remove many nucleotides upon each binding event. By contrast, LASV NP-ExoN exhibits a less processive behavior and removes fewer nucleotides upon each binding event. The two enzymes also show a difference in the minimal dsRNA length for efficient degradation. We show that nsp14/10 associates with a dsRNA substrate at a faster rate than NP-ExoN and correspondingly has a higher affinity for dsRNA, which is likely to account for the higher processivity. These observations help to understand the biological functions of the viral ExoN enzymes and underscore roles for nsp14/10 beyond proofreading.

**IMPORTANCE:**

Coronaviruses such as SARS-CoV-2 and arenaviruses such as Lassa virus (LASV) share a structurally similar exoribonuclease (ExoN) domain as part of the viral non-structural protein 14 (nsp14) and nucleoprotein (NP), respectively, which degrades RNA strands from the 3′ terminus. The ExoN activity of coronavirus nsp14 is known for its role in proofreading, where it removes nucleotides misincorporated during viral RNA synthesis, whereas that of arenavirus NP is known for its importance in suppressing the host’s immune response by degrading immunostimulatory RNAs. In this study, we show that SARS-CoV-2 ExoN is highly processive and removes many nucleotides upon each binding event, whereas LASV NP ExoN is less processive and removes fewer nucleotides at a time. We further show that the contrasting behaviors are attributable to distinct RNA-binding kinetics of these two viral enzymes. These observations help to understand the biological roles of the viral ExoN enzymes and suggest their pleiotropic functions.

## INTRODUCTION

The replication of RNA viruses depends not only on efficient synthesis but also on the degradation of RNA. While all known RNA viruses encode an RNA-directed RNA polymerase (RdRp) or a reverse transcriptase (1, 2), many of these viruses rely on the activity of host exoribonucleases for the degradation of viral or host RNAs to facilitate virus genome replication, manipulation of host physiology, and evasion of antiviral immunity (3–5). However, two distinct families of RNA viruses, namely, nidoviruses and arenaviruses, encode their own 3′–5′ exoribonucleases (ExoNs) that play essential roles in replication (6, 7), maintaining viral genomic fidelity (8), and suppressing the host immune response (9).

Coronaviruses (CoVs) are among the nidovirus family and feature large RNA genomes of approximately 30 kb. The ExoN domain in the N-terminus of CoV non-structural protein 14 (nsp14) supports the replication of this large genome through proofreading during RNA synthesis by the low-fidelity viral RdRp (10–16). In addition to removing misincorporated nucleotides during RNA synthesis, the nsp14 ExoN activity has also been shown to be required for various aspects of RNA metabolism, including the generation of subgenomic RNAs (6), viral RNA recombination (17), shutdown of host translations (18), and evasion of host innate immune responses (19–21). Arenaviruses have smaller (~11 kb) and segmented ambisense genomes that encode only four proteins. One of these four viral proteins, nucleoprotein (NP), consists of an N-terminal ssRNA-binding domain responsible for ribonucleoprotein (RNP) complex formation in genome packaging and the C-terminal ExoN domain (22–24). Arenavirus NP plays a significant role in altering the host immune response by inhibiting the IRF-3 activation pathway and nuclear factor kappa B signaling (25–30). The ExoN domain of NP is responsible for degrading virus-derived immunostimulatory RNAs to evade host immunity (31, 32) by interferon suppression and modest reduction of viral RNA replication (33), along with its role in maintaining the integrity of the viral genome (34). Arenavirus NP suppresses type I interferon response in macrophages and dendritic cells (35) and weakens natural killer cell responses *in vitro* (36). However, despite the importance of the ExoN activities in both CoV and arenavirus replication, the full extent of physiological RNA targets and the mechanisms by which they enable virus replication are not fully understood.

**Fig 1:**
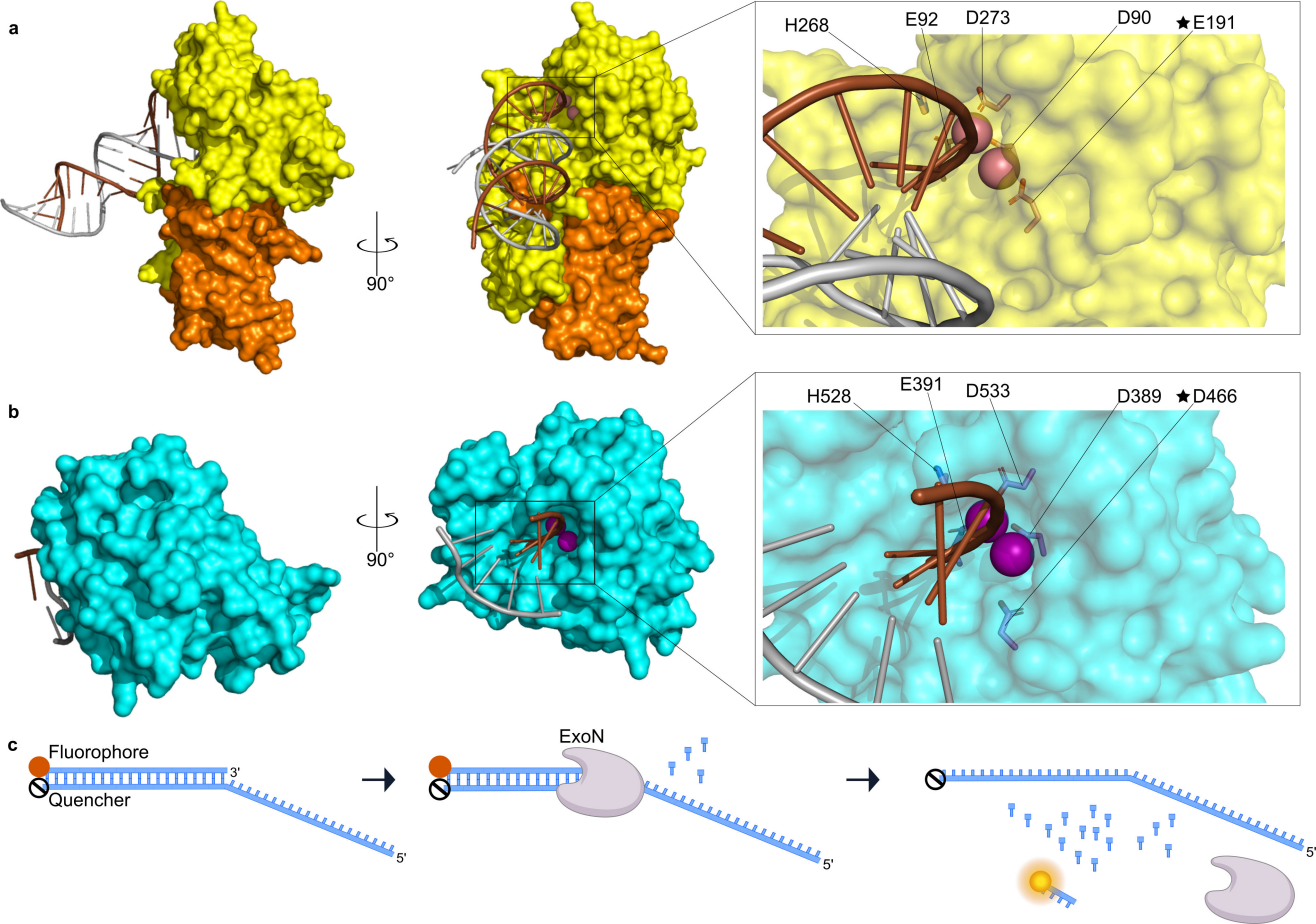
Structures of SARS-CoV-2 and LASV exoribonucleases and the real-time kinetic enzyme assay principle. (**a**) Cryo-EM structure of SARS-CoV-2 nsp14/10 complex bound to dsRNA (PDB ID: 7n0b) (38). (**b**) Crystal structure of Lassa virus nucleoprotein C-terminal domain (PDB ID: 4gv9) bound to dsRNA (37). The proteins are shown in molecular surface, where SARS-CoV-2 nsp14 is shown in yellow; nsp10 is shown in orange; and Lassa virus nucleoprotein is shown in cyan. The N7-methyltransferase domain of SARS-CoV-2 nsp14 is omitted in panel a. The active site residues are shown in stick and colored in pink and purple for SARS-CoV-2 and LASV, respectively. The active form of SARS-CoV-2 nsp14/10 is shown, where the catalytic magnesium ions are replaced with calcium, shown in pink spheres. Manganese is the catalytic metal for LASV, shown in purple spheres. The scissile strand of dsRNA is shown in brown, and the non-scissile strand is in light gray. Catalytic residues in the DEDDh motif that differ between the two enzymes are marked with black stars. Molecular representations were rendered using PyMOL (https://www.pymol.org/). (**c**) Cartoon representation of ExoN-mediated RNA degradation in a fluorescence-based assay. The top strand is a 5′-labeled 20-nucleotide (nt) RNA oligo annealed to a 40-nt complementary RNA strand with the respective quencher on the 3′ terminus (LS2U_RNA20 and LS15A, Table 1). Enzyme activity was studied either by plate reader scans in real-time or by time course samples analyzed on polyacrylamide gel.

Both the CoV and arenavirus ExoN domains are divalent metal-dependent exonucleases featuring a conserved set of metal ion-coordinating residues designated the DEDDh motif (Fig. S1), also found in many cellular RNA and DNA nucleases (37–39). Structural studies have revealed similar tertiary structures and RNA substrate binding mechanisms of the CoV and arenavirus ExoN, confirming their evolutionary relationship (37, 38, 40). Both ExoN enzymes show a strong preference for double-stranded RNA (dsRNA) (6, 10, 31), which is explained by similar interactions with the scissile strand in ExoN-dsRNA cryo-EM or crystal structures (Fig. 1a and b). However, these structurally related viral ExoN enzymes also have key differences, including the strict requirement for Mg^2+^ by CoV and Mn^2+^ by arenavirus ExoN and the unique requirement for another viral protein, nsp10, by CoV nsp14 ExoN for its activity (10, 24, 31, 41–43). In this study, we developed a quantitative assay and examined the biochemical activities of these two viral ExoN enzymes to gain insight into their respective biological functions. Our comparative analyses highlight contrasting behaviors of the two proteins, which may underlie distinct roles of each viral ExoN enzyme in virus replication.

## RESULTS

### Real-time assay for ExoN enzyme kinetics

To examine the biochemical properties of viral ExoN enzymes, we developed a real-time fluorescence-based assay similar to those previously reported (41, 44, 45). Our assay uses a 5′ Cy3-labeled 20-nucleotide (nt) RNA oligo annealed to a 40-nt complementary RNA strand with a dark quencher on the 3′ terminus (LS2U_RNA20_5′Cy3 and LS15A_IowaBlackRQ; Table 1; Fig. 1c). The 5′ Cy3 and 3′ IowaBlack RQ quencher gave the best signal-to-noise ratio in a preliminary fluorophore optimization screen, which was the basis behind choosing this fluorophore and quencher pair (data not shown). These two RNA oligos form a 20-bp double-stranded RNA with a 20-nt 5′-overhang, mimicking an RdRp-mediated extension intermediate used in previous CoV studies (14, 42). The Cy3 fluorescence signal is quenched in this state due to the physical proximity of the fluorophore and the quencher. The fluorescently labeled strand has a free 3′-OH terminus, whereas degradation of the complementary strand is blocked by its 3′ modification. Upon addition of ExoN and incubation at 26°C, the fluorescently labeled strand is degraded exonucleolytically, eventually to a small RNA fragment too short to remain base-paired to the quencher strand. This results in the release of the fluorophore and liberation of fluorescence over time, allowing for monitoring of the kinetics of ExoN-mediated RNA degradation.

**TABLE 1:** Oligonucleotides used in this study

Name	Sequence^*a*^
LS2U_RNA10_5′Cy3	/5Cy3/rGrUrCrArUrUrCrUrCrC
LS2U_RNA20_5′Cy3	/5Cy3/rGrUrCrArUrUrCrUrCrCrUrArArGrArArGrCrUrU
LS2U_RNA20_5′Cy5	/5Cy5/rGrUrCrArUrUrCrUrCrCrUrArArGrArArGrCrUrU
LS2U_RNA20_5′FAM	/56-FAM/rGrUrCrArUrUrCrUrCrCrUrArArGrArArGrCrUrU
LS2U_RNA20_5′Biotin	/5Biosg/rGrUrCrArUrUrCrUrCrCrUrArArGrArArGrCrUrU
LS2U_RNA30_5′Cy3	/5Cy3/rGrUrCrArUrUrCrUrCrCrUrArArGrArArGrCrUrUrGrUrArArArArUrCrArC
LS15A_RNA	rCrUrArUrCrCrCrCrArUrGrUrGrArUrUrUrUrArCrArArGrCrUrUrCrUrUrArGrGrArGrArArUrGrArC
LS15A_RNA-ddC	rCrUrArUrCrCrCrCrArUrGrUrGrArUrUrUrUrArCrArArGrCrUrUrCrUrUrArGrGrArGrArArUrGrA/3ddC/
LS15A_IowaBlackFQ	rCrUrArUrCrCrCrCrArUrGrUrGrArUrUrUrUrArCrArArGrCrUrUrCrUrUrArGrGrArGrArArUrGrArC/3IABkFQ/
LS15A_IowaBlackRQ	rCrUrArUrCrCrCrCrArUrGrUrGrArUrUrUrUrArCrArArGrCrUrUrCrUrUrArGrGrArGrArArUrGrArC/3IAbRQSp/
U20_RNA_5'FAM	/56-FAM/rUrUrUrUrUrUrUrUrUrUrUrUrUrUrUrUrUrUrUrU
A30_RNA	rArArArArArArArArArArArArArArArArArArArArArArArArArArArArArA

^*a*^ r denotes ribonucleotide.

### SARS-CoV-2 and Lassa virus ExoNs show contrasting behaviors

**Fig 2:**
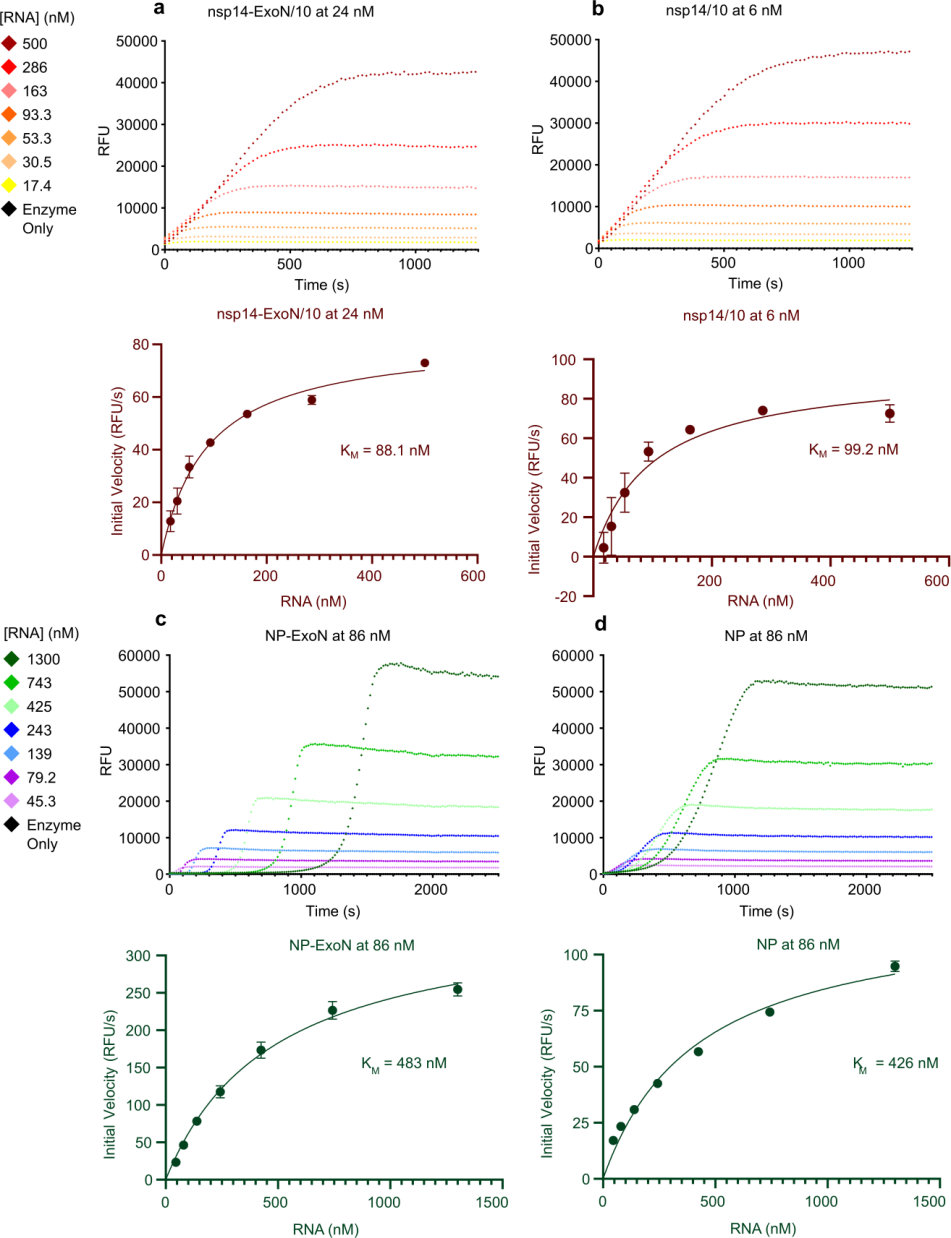
Real-time fluorescence traces and *K*_M_ analyses comparing exonuclease domain-only and full-length versions of SARS-CoV-2 nsp14/10 and Lassa virus nucleoprotein (NP) in dsRNA titration. Raw real-time fluorescence traces of nsp14-ExoN/10 (**a**) and nsp14/10 (**b**), where enzyme concentration is fixed and dsRNA is titrated (LS2U_RNA20_5′Cy3 annealed to LS15A_IowaBlackRQ; Table 1). The corresponding curves (maroon) show fitting of raw data to the Michaelis-Menten model (*R*^2^ of 0.973 and 0.888, respectively). The *K*_M_ values were determined to be 88.1 nM with 95% CI [71.9, 108] and 99.2 nM with 95% CI [64.3, 154] for nsp14-ExoN/10 and nsp14/10, respectively. As dsRNA concentration changes, both exonuclease domain-only and full-length versions of SARS-CoV-2 nsp14/10 are affected primarily in the slopes of their raw kinetic traces. This is in contrast to NP-ExoN (**c**) and NP (**d**), for which the initial lag phase is extended as the dsRNA concentration is increased. Michaelis-Menten plots for the LASV enzymes are shown in green (*R*^2^ = 0.989 and 0.981 for NP-ExoN and NP, respectively). The *K*_M_ values were determined to be 483 nM with 95% CI [411, 570] and 426 nM with 95% CI [346, 527] for NP-ExoN and NP, respectively.

Using the real-time assay, we first examined the activities of both the full-length and an ExoN domain fragment of SARS-CoV-2 nsp14/10 and Lassa virus (LASV) NP. As expected, all four enzymes degraded the fluorogenic dsRNA substrate to yield a time-dependent increase in Cy3 fluorescence. However, reaction kinetics of the proteins from the two different viruses showed distinct characteristics. In dsRNA titration experiments, the N-terminal ExoN fragment of nsp14 in complex with nsp10 (nsp14-ExoN/10) generated an approximately linear increase of fluorescence intensity over time, and varying the substrate concentration against a fixed concentration of enzyme mainly affected the slope and final plateau level of the kinetic traces (Fig. 2a; Fig. S2a and S4a). The full-length nsp14/10 complex also showed a similar behavior (Fig. 2b; Fig. S2a and S4b). In contrast, increasing the dsRNA concentration against the C-terminal ExoN domain fragment of NP (NP-ExoN) resulted in biphasic kinetic traces featuring an initial lag phase before the onset of fluorescence intensity increase (Fig. 2c; Fig. S3a and S4c). Full-length NP also showed the same extended lag phase as NP-ExoN, though not as striking (Fig. 2d; Fig. S3a and S4d). Kinetic parameters were determined from these experiments based on the maximal rate of fluorescence increase as a function of RNA concentration. The *K*_M_ values were 88.1 and 99.2 nM for nsp14-ExoN/10 and nsp14/10, respectively (Fig. 2a and b). For NP-ExoN and full-length NP, the *K*_M_ values were 483 and 426 nM, respectively (Fig. 2c and d). The *K*_M_ values show that the ExoN domain alone and the full-length protein have comparable apparent affinities for dsRNA for both viral enzymes and that SARS-CoV-2 nsp14/10 has a higher apparent affinity for dsRNA compared to LASV NP.

**Fig 3:**
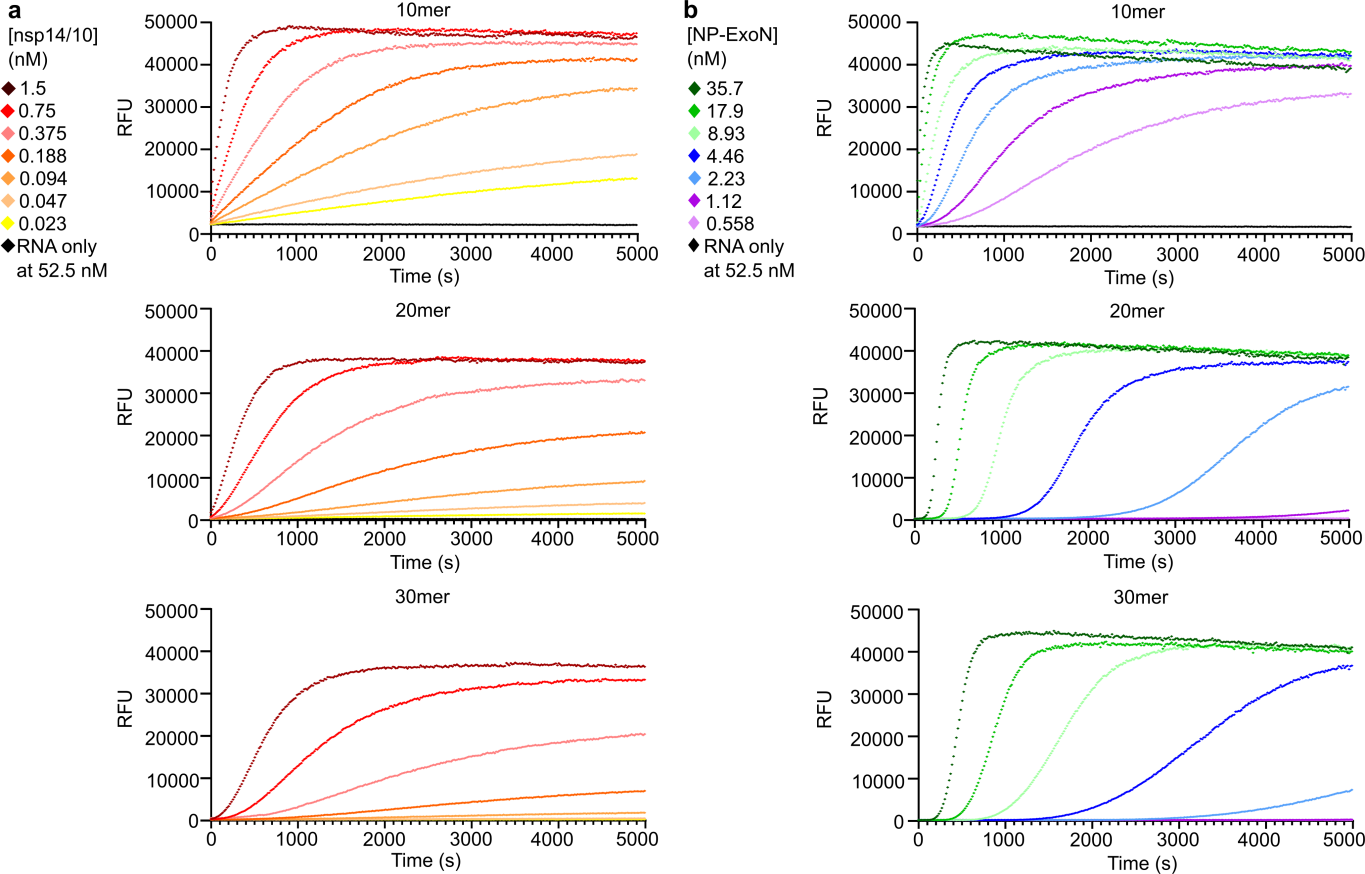
Varying RNA length highlights differences in exonuclease activity between nsp14/10 and NP-ExoN. Real-time fluorescence-based enzyme degradation experiments tested against varying top-strand lengths of dsRNA substrates (LS2U_RNA10_5′Cy3, LS2U_RNA20_5′Cy3, and LS2U_RNA30_5′Cy3; Table 1). (**a**) nsp14/10 titration kinetic traces show a linear increase of fluorescence over time, and lengthening RNA primarily led to a slower fluorescence increase. (**b**) NP-ExoN enzyme titration kinetic traces, by contrast, show characteristic biphasic slopes, and lengthening RNA primarily led to a longer initial lag phase. The kinetic traces above are representative of experiments performed in triplicate (Fig. S5 and S6).

Similarly, in an enzyme titration against a fixed concentration of the same dsRNA used above, lowering the nsp14/10 concentration led to a gradual decrease in the slope of the kinetic traces (Fig. 3a [middle] and Fig. S5 [middle]). Nsp14-ExoN/10 showed a very similar concentration dependence pattern (Fig. S2b). By contrast, lowering the NP-ExoN concentration against a fixed concentration of dsRNA led to a progressive lengthening of the lag phase, in addition to the reduction of slopes in the second phase (Fig. 3b [middle] and Fig. S6 [middle]). We chose to directly compare nsp14/10 and NP-ExoN in further kinetic studies due to NP-ExoN’s more pronounced extension of the lag phase (Fig. 2c and d) and the superior activity displayed by nsp14/10 compared to nsp14-ExoN/10 (Fig. 3a [middle]; Fig. S2b, Fig. S5 [middle], and Fig. S7a). NP and NP-ExoN’s activities were also compared in the same enzyme titration scheme, and both enzymes featured biphasic behavior despite NP-ExoN displaying superior activity (Fig. 3b [middle]; Fig. S6 [middle], and Fig. S7b).

### Processive vs non-processive RNA degradation

The pronounced biphasic kinetics observed for NP-ExoN, with progressively longer lag time in the presence of a greater fold-molar excess of dsRNA over the enzyme, can be explained if we assume that NP-ExoN removes a smaller number of nucleotides upon each binding event. Then, the initial lag phase represents the time during which RNA substrates are gradually degraded but are still long enough to be quenched. As the enzyme distributively acts on many RNA molecules, higher RNA concentration leads to a longer lag phase (Fig. 2c). Once the RNA pool is degraded past a critical point, it dissociates from the complementary strand to generate a fluorescence signal. In this scenario, the slope of a kinetic trace reflects the rate at which the last nucleotide is removed before strand dissociation. On the other hand, nsp14/10 generated more linear kinetic traces with a much less pronounced initial lag phase, even in the presence of a large molar excess of dsRNA over the enzyme (Fig. 2b). This suggests that nsp14/10 behaves more processively than NP-ExoN and removes a greater number of nucleotides upon each binding event, which causes the ensemble of RNA substrates to be degraded more sequentially than in parallel. The slope of a kinetic trace, in this case, would depend on the rate at which the enzyme degrades through each substrate to reach the critical point and induce strand dissociation, including the rate of removal of the last nucleotide(s).

### RNA length dependence

**Fig 4:**
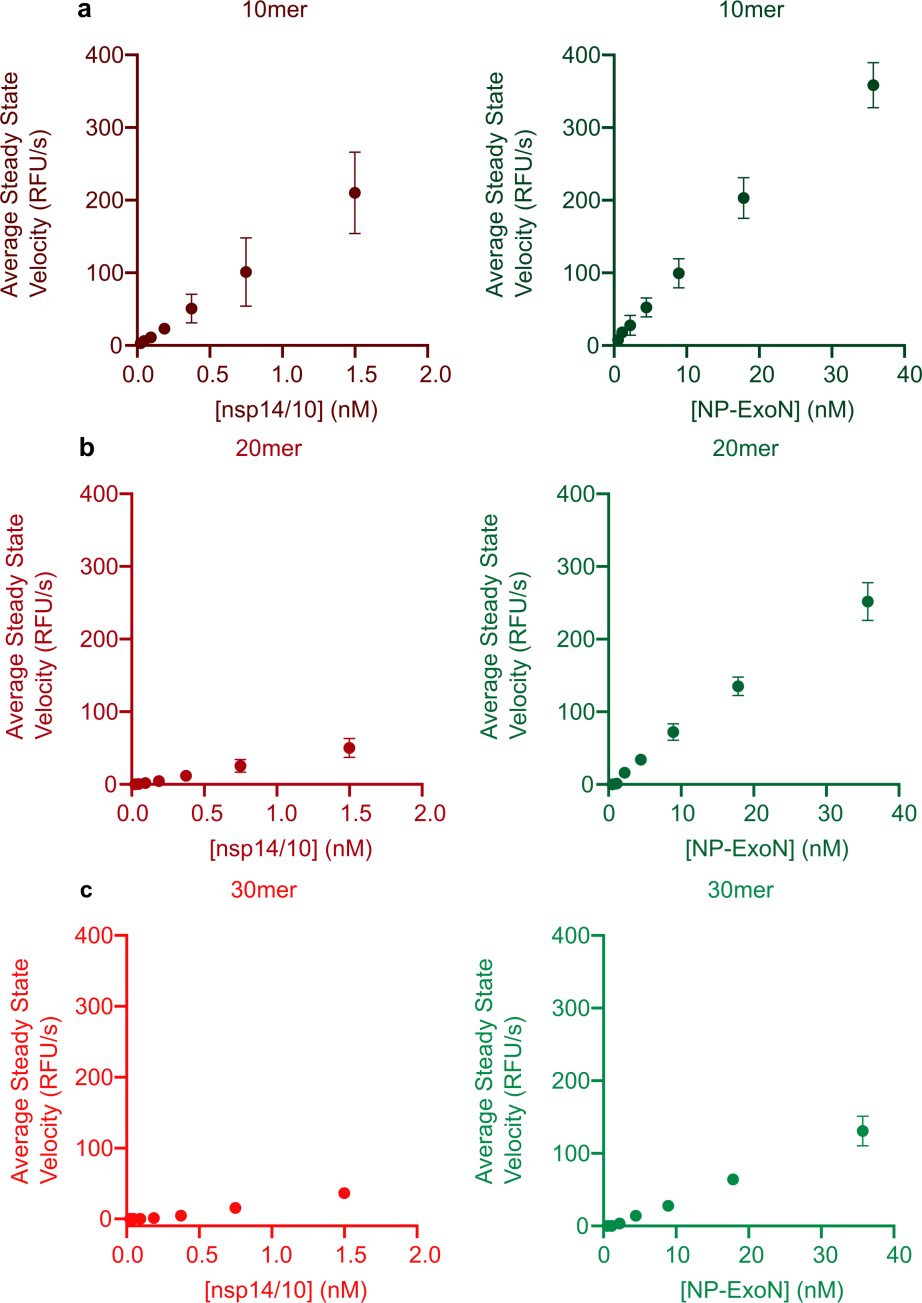
Comparison of nsp14/10 and NP-ExoN kinetic slope quantitation. Analyses of data in Fig. 3 and Fig. S5 and S6 (*n* = 3), where the top strand of dsRNA was 10 nt (**a**), 20 nt (**b**), or 30 nt (**c**). Extending the top strand of the dsRNA substrate results in a decrease in slopes for both nsp14/10 and NP-ExoN. The extent to which steady-state velocity decreases is greater in the case of nsp14/10. When the substrate extends from 10 to 30 bp, the slopes for nsp14/10 decrease 17.4-fold, whereas a more modest decrease of 4.81-fold is observed for NP-ExoN. The two lowest enzyme conditions were left out of the average due to the longer dsRNA slopes being too shallow to quantify.

To test the hypothesis regarding the processive and non-processive (distributive) behaviors of nsp14/10 and NP-ExoN, respectively, we compared the two enzymes’ activities on dsRNA substrates with different lengths. We reasoned that extending the top strand of the dsRNA substrate (LS2U_RNA30_5′Cy3, Table 1) would cause the enzymes to take a longer time to degrade the substrate down to the critical length and thus would reduce the slope of kinetic traces for a highly processive enzyme, whereas it would most significantly prolong the initial lag phase rather than the slope for a non-processive enzyme. As expected, the slopes of the kinetic traces for nsp14/10 became progressively smaller as the dsRNA substrate length increased from 10 to 30 bp (Fig. 3a; Fig. S5). Compared to the slopes on 10 bp, the maximum slopes decreased by 4.81-fold on average for 20 bp and 17.4-fold for 30 bp (Fig. 4a through c). On the other hand, the reduction of slopes was much more modest for NP-ExoN (Fig. 3b; Fig. S6): 1.52-fold for 20 bp and 4.17-fold for 30 bp, compared to the slopes on 10 bp (Fig. 4a through c). Instead, the kinetic traces generated by NP-ExoN were most noticeably affected in the length of their initial lag phase. As expected, the initial lag time in NP-ExoN’s characteristic biphasic slope became longer as the RNA substrate length was increased or the enzyme concentration was decreased (Fig. 4a through c; Fig. S8). In contrast, the kinetic traces generated by nsp14/10 lack a substantial lag phase across all enzyme titrations due to the linear fluorescence increase (Fig. 3a; Fig. S5). Taken together, these observations support our hypothesis that nsp14/10 operates more processively than NP-ExoN.

### Gel-based analysis

**Fig 5:**
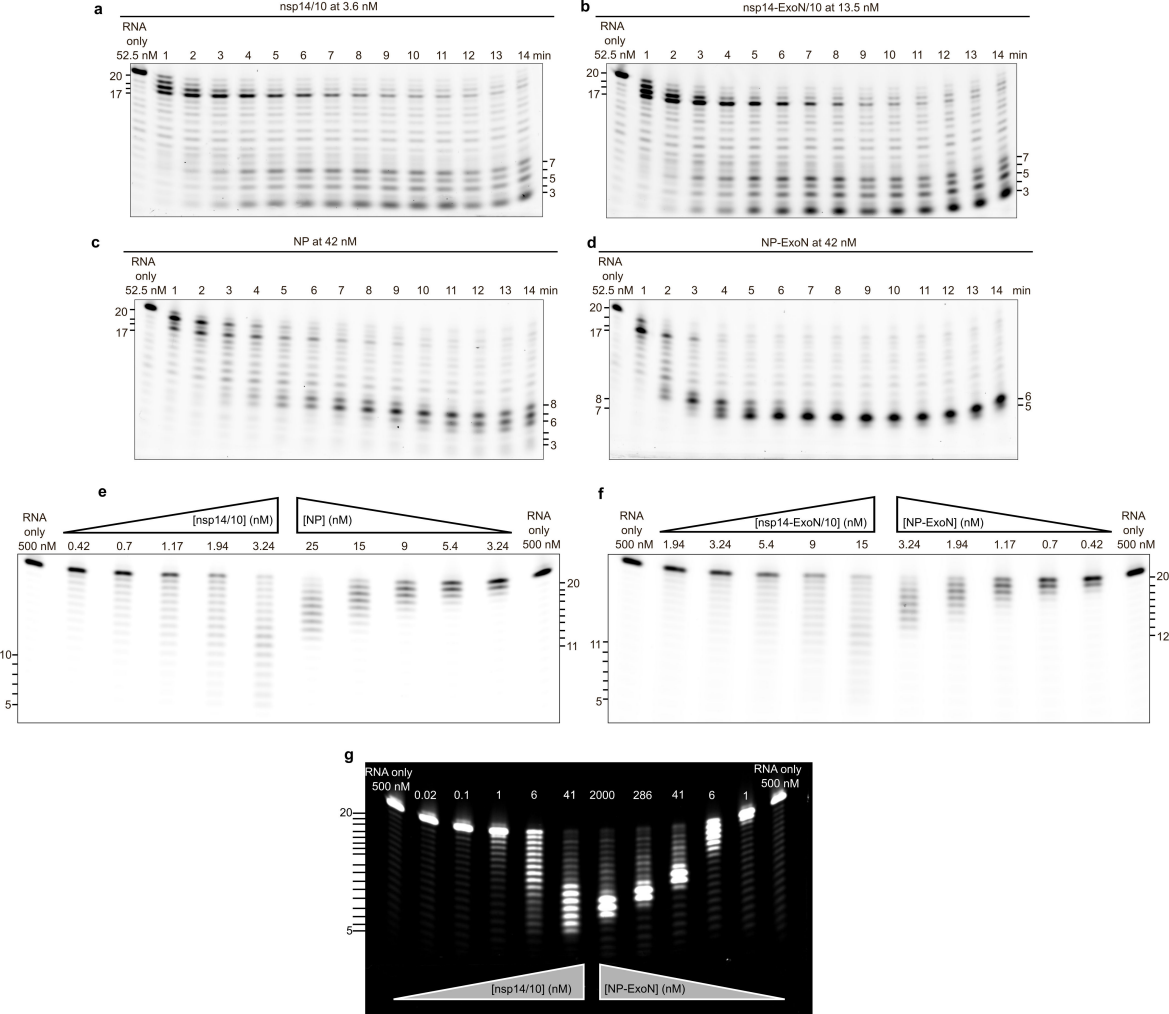
Time-resolved product distribution, single-hit endpoint, and spiked enzyme concentration endpoint gel analyses show differences between SARS-CoV-2 and LASV exonuclease activities. (**a–d**) Time course gel experiments assessing degradation of fluorescently labeled RNA oligonucleotides into shorter RNA fragments, where substrate and nsp14/10 (**a**), nsp14-ExoN/10 (**b**), NP (**c**), or NP-ExoN (**d**) were held constant. The dsRNA substrate used in this experiment was the same as the oligo used in Fig. 2, except with a different fluorophore. (**e and f**) Single-hit endpoint experiments of the full-length (**e**) and exonuclease versions (**f**) of both viral proteins, where homopolymer dsRNA (U20_RNA_5′FAM annealed to A30_RNA; Table 1) was in excess and held constant (500 nM) against a titration of each respective enzyme. (**g**) Gel experiments where homopolymer dsRNA was held constant against nsp14/10 or NP-ExoN, and the highest enzyme concentration was spiked to further observe RNA degradation into shorter fragments. The data above are representative of duplicated experiments.

To complement the real-time assay above, we further compared the two enzymes’ activities in a gel-based assay. Denaturing gel electrophoresis of the same 20 bp dsRNA substrate as that used in the real-time assay (LS2U_RNA20_5′FAM annealed to LS15A_IowaBlackFQ; Table 1) was performed to monitor time-resolved product distributions, where both substrate and enzyme were held constant (Fig. 5a through d). The 6-FAM fluorophore (instead of Cy3) on the 5′ end of the scissile strand was used in gel-based assays for the best results in imaging.

Both full-length and ExoN domain-only versions of the viral enzymes showed the accumulation of a degradation intermediate corresponding to an RNA 3 nt shorter than the starting substrate, suggesting a common roadblock. Nonetheless, the reactions with nsp14/10 and nsp14-ExoN/10 showed an RNA species that has been degraded to a point where it cannot be processed further (3-mer), even in time points as early as 2 min against a large molar excess of RNA (Fig. 5a and b).

On the other hand, the shortest products produced by NP and NP-ExoN were 3 and 5 nt, respectively, although the majority of the products remained 6 nt for both (Fig. 5c and d). Under the assay condition used, either with magnesium or manganese, the melting temperature (*T*_*m*_) predicted by the OligoAnalyzer tool (46) (Integrated DNA Technologies) drops below 10°C once the substrate RNA is degraded to 8 nt. Because the gel-based assays were performed at room temperature (~20°C), it is possible that the presence of the enzyme stabilized the dsRNA substrate to allow continued degradation past the critical length for strand separation or that the products shorter than 7 nt were generated due to the enzyme’s activity on ssRNA. Regardless, shorter RNA products are seen earlier in the time course for nsp14/10 and nsp14-ExoN/10, as compared to NP and NP-ExoN. These data are consistent with the higher processivity of the SARS-CoV-2 than LASV enzymes, which underlies the distinct biphasic kinetics in the real-time fluorescence signals.

As we showed previously (42), nsp14/10 degrades poly-U RNA only in the presence of a complementary poly-A RNA. To further investigate the processivity difference between SARS-CoV-2 and LASV, an exonuclease activity assay in single-hit conditions was performed using excess 5′ FAM-labeled poly-U20 RNA in the presence of poly-A30 (Table 1) incubated with low concentrations of nsp14/10, nsp14-ExoN/10, NP, or NP-ExoN. The RNA products analyzed on a denaturing polyacrylamide gel showed distinct ladder patterns consisting of different distributions of RNA lengths (Fig. 5e and f). Full-length versions and exonuclease versions of the two viral enzymes were loaded side-by-side for ease of comparison.

The reactions with the lowest concentration of NP (3.24 nM) or NP-ExoN (0.42 nM) against 500 nM RNA showed mostly the intact 20-mer RNA with some 19-mer, and a small amount of 18-mer product. The reactions with a slightly higher NP (5.4 nM) or NP-ExoN (0.70 nM) concentration generated a distribution of 17- to 20-mers, with the 19- and 20-mers still being the most abundant. The RNA products became incrementally shorter as the LASV enzyme concentrations were increased, and the highest NP (25 nM) or NP-ExoN (3.24 nM) concentration generated products centered around 15- to 16-mers, with the intact 20-mer being mostly depleted. The tight distribution and gradual shortening of RNA are consistent with the non-processive activity of LASV NP and NP-ExoN.

By contrast, the SARS-CoV-2 enzymes showed a distinct dose response pattern, in which the intact 20-mer RNA persists better while shorter products are generated at the same time (Fig. 5e and f). The reactions with the lowest concentrations of nsp14/10 (0.42 nM) or nsp14-ExoN/10 (1.94 nM) against 500 nM RNA showed mostly the intact 20-mer RNA band, with a faint ladder of RNA products underneath. The reactions with higher enzyme concentrations generated wider distributions of RNA products, including those smaller than 10 nt, while still retaining a significant amount of the 20-mer. The reactions with the highest nsp14/10 (3.24 nM) or nsp14-ExoN/10 (15.0 nM) concentrations generated products down to a 5-mer but notably still containing some intact 20-mer RNA. Further increasing the enzyme concentration for both SARS-CoV-2 and LASV enzymes did not generate products shorter than 5-mer (Fig. 5g), and the products generated with 300 nM enzyme ranged from 5- to 8-mers (Fig. S9). This is likely to represent the limit of ExoN-mediated degradation due to the stability of poly-U/A dsRNA, as the *T*_*m*_ predicted by the OligoAnalyzer tool (46) for a 9 bp poly-U/A is already well below 10°C. The observed behavior is consistent with the processive activity of SARS-CoV-2 nsp14/10.

### RNA-binding affinities

**Fig 6:**
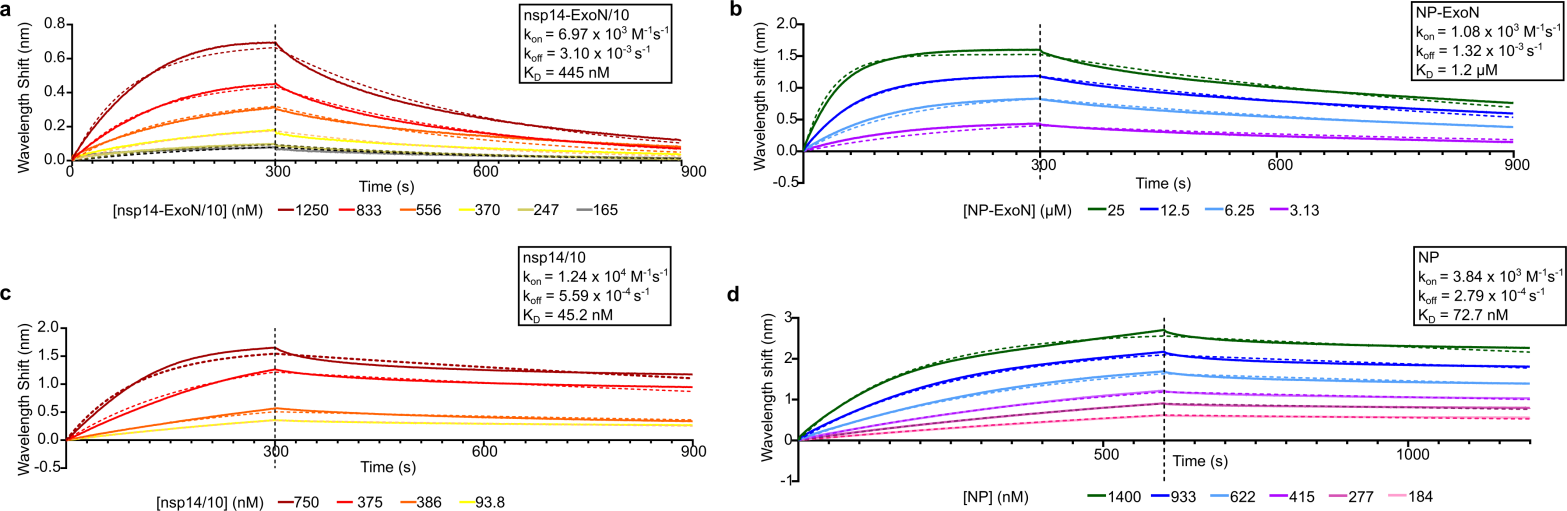
Comparing dsRNA binding of viral enzymes by biolayer interferometry (BLI). BLI sensograms showing the association and dissociation kinetics of nsp14-ExoN/10 (**a**), NP-ExoN (**b**), nsp14/10 (**c**), and NP (**d**), where the titrated enzyme is in excess of the loaded dsRNA (LS2U_RNA20_5′Biotin and LS15A_RNA-ddC, Table 1). Best-fit curves to the single-site binding model are shown as dotted lines (*R*^2^ of 0.992 for both SARS-CoV-2 enzymes and *R*^2^ of 0.990 and 0.998 for NP-ExoN and NP, respectively). NP-ExoN’s *K*_D_ is 26.5-fold greater than that for nsp14/10, where the higher affinity of nsp14/10 for dsRNA is primarily due to the greater rate constant of association.

A possible explanation for the higher processivity of SARS-CoV-2 nsp14/10 is that its ExoN domain has a higher affinity for dsRNA than that of LASV NP, as suggested by the *K*_M_ values derived from the real-time assays. To test this hypothesis, we used biolayer interferometry (BLI) and examined the binding of nsp14-ExoN/10 and NP-ExoN to the dsRNA substrate with a 5′-overhang (LS2U_RNA20_5′Biotin annealed to LS15A_RNA-ddC [Table 1]) (Fig. 6a and b). We also analyzed the binding of the full-length proteins, which have a C-terminal guanine-N7 methyltransferase domain connected to the ExoN domain of nsp14 or an N-terminal ssRNA binding domain for LASV NP (Fig. 6c and d). Supporting our hypothesis, nsp14-ExoN/10 showed a higher affinity (Fig. 6a; *K*_D_ = 445 nM, *k*_on_ = 6.97 × 10^3^ M^−1^s^−1^, *k*_off_ = 3.1 × 10^−3^ s^−1^) than NP-ExoN (Fig. 6b; *K*_D_ = 1.2 µM, *k*_on_ = 1.08 × 10^3^ M^−1^s^−1^, *k*_off_ = 1.32 × 10^−3^ s^−1^). Notably, nsp14-ExoN/10 is faster in both association and dissociation kinetics: the ~2.7-fold higher affinity results from a ~6.5-fold greater *k*_on_ and a ~2.3-fold greater *k*_off_. The full-length counterparts of these enzymes bound more tightly to dsRNA, where nsp14/10 (Fig. 6c; *K*_D_ = 45.2 nM, *k*_on_ = 1.24 × 10^4^ M^−1^s^−1^, *k*_off_ = 5.59 × 10^−4^ s^−1^) again showed a slightly higher affinity than NP (Fig. 6d; *K*_D_ = 72.7 nM, *k*_on_ = 3.84 × 10^3^ M^−1^s^−1^, *k*_off_ = 2.79 × 10^−4^ s^−1^) with greater *k*_on_ and *k*_off_. Although the comparison between full-length nsp14/10 and NP is complicated by possible contributions of the non-ExoN domain of each protein and that NP forms a stable homotrimer (47), which would increase apparent affinity for the RNA ligand immobilized on the sensor chip surface due to higher avidity, the conserved trend of the SARS-CoV-2 enzymes showing higher affinity and faster association/dissociation is interesting and may underlie their processive behavior. In particular, the faster association would allow nsp14/10 to quickly rebind RNA after every catalytic cycle for hydrolyzing the next nucleotide, which may lead to higher processivity.

### Mutational analysis

**Fig 7:**
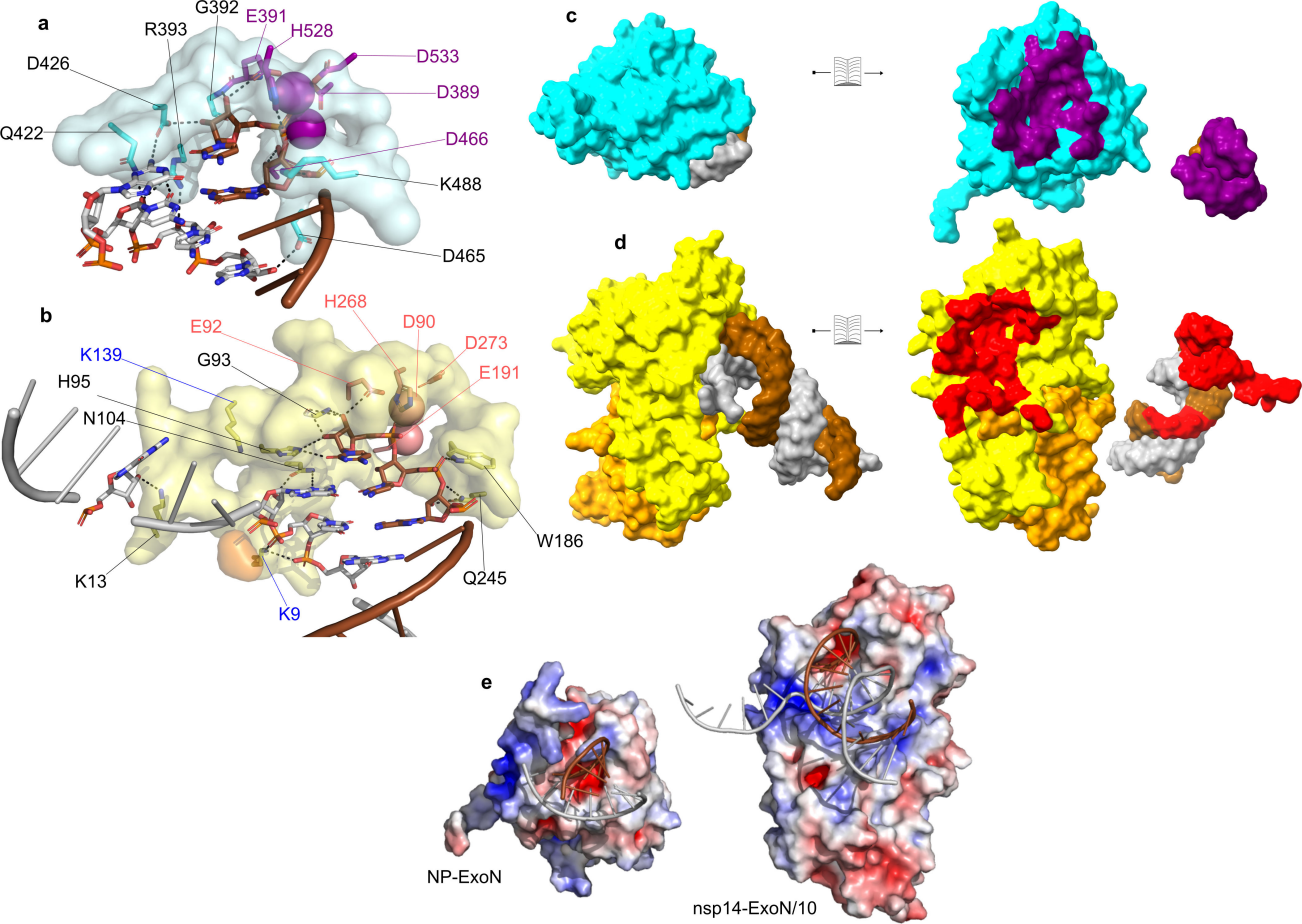
Analyses of ExoN-dsRNA polar contacts, buried surface area, and electrostatics. (**a and b**) Close-up view of the NP-ExoN (PDB: 4gv9) (37) (**a**) and nsp14-ExoN/10 (PDB: 7n0d) (38) (**b**) active sites bound to dsRNA. Hydrogen bonds or salt bridges are indicated by black dashed lines, where the distance cutoff is 3.5 Å. Protein within 5 Å of dsRNA is shown in a transparent molecular surface, with active site residues and residues contacting dsRNA shown in sticks. Active site residues are colored purple and pink for NP-ExoN and nsp14-ExoN/10, respectively. Nsp14-ExoN/10 residues that were investigated in mutational studies are labeled in blue. The catalytically inactive nsp14-ExoN(E191A)/10-dsRNA cryo-EM structure is shown (PDB: 7n0d) (38) due to its high resolution for accurate representation of protein-RNA interactions, except that the metal ions are from the lower-resolution active enzyme structure (38), and A191 is changed back to E191 for display purposes. The exonucleases contact the scissile strand in similar manners, where a conserved glycine interacts with the terminal 3′-OH group (G392 and G93 for NP-ExoN and nsp14-ExoN/10, respectively). However, the 3′ terminal nucleotide bound to nsp14-ExoN/10 is involved in twice as many contacts. (**c and d**) Buried surface area of NP-ExoN (**c**) and nsp14-ExoN/10 (**d**) shown in purple (1240 Å^2^) and red (1756 Å^2^), respectively. NP-ExoN is in cyan; nsp14-ExoN is in yellow; and nsp10 is in orange. (**e**) ExoN-dsRNA complexes with molecular surfaces colored according to the electrostatic potential (−5 kT/e in red to + 5 kT/e in blue) as calculated by APBS (48). This figure was generated with the NP-ExoN structure with a basic loop (40) (PDB: 4fvu). Polar interactions, buried surface area calculations, and electrostatic surface representations were made using PyMOL software. Buried surface area graphics were rendered using UCSF ChimeraX (49).

**Fig 8:**
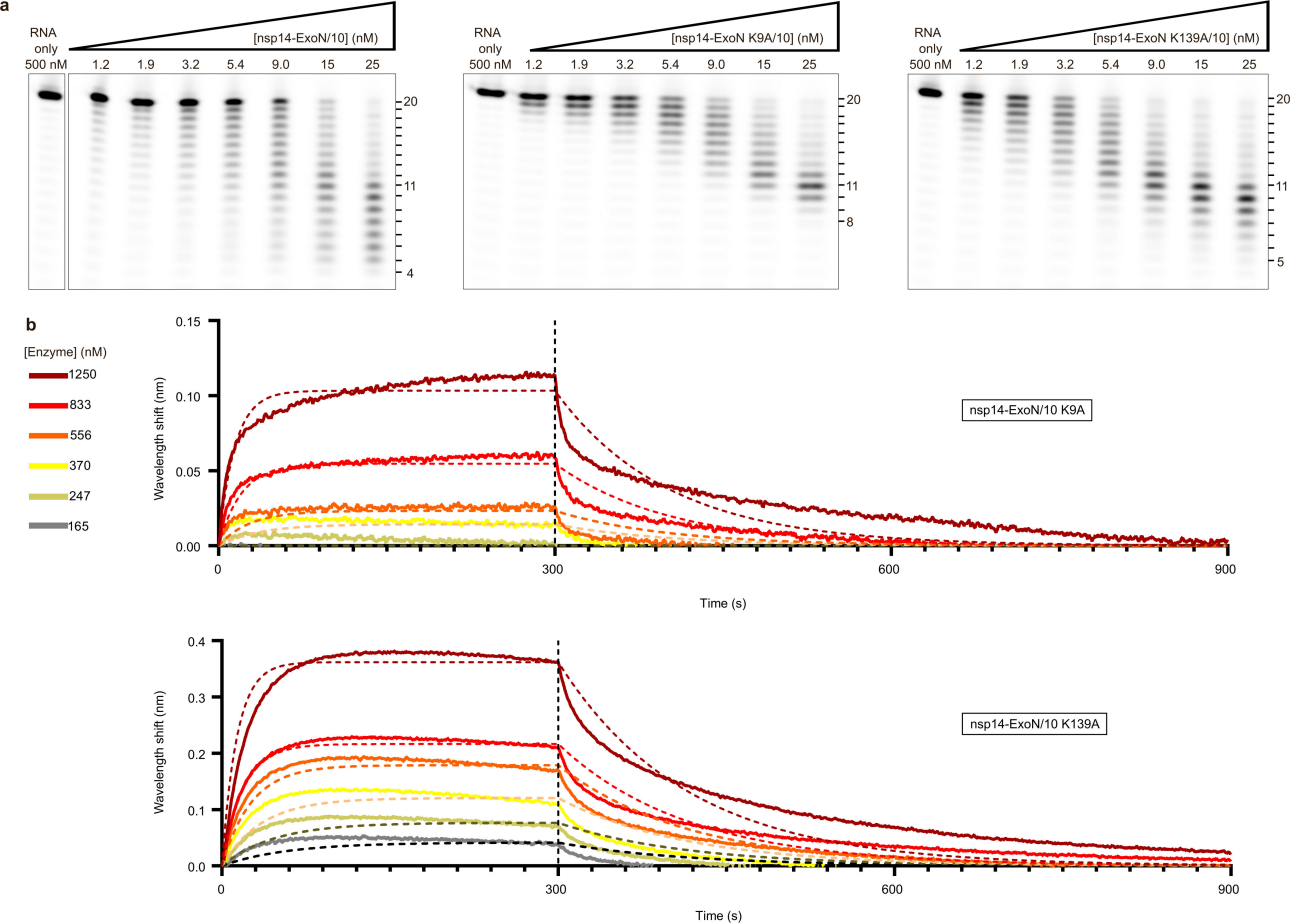
Mutational analysis of RNA-contacting and proximal lysine residues. (**a**) Single-hit endpoint analyses. Wild-type (WT) or mutants of nsp14-ExoN/10 were titrated against a fixed concentration of dsRNA (same substrate as in Fig. 5e through g) for comparison. Degradation of the 20-nt RNA substrate was observed on gel. K9 directly contacts the dsRNA backbone, whereas K139 is in a proximal basic patch potentially involved in positioning dsRNA to the active site (42) (Fig. 7b and e). Mutating either of these residues to alanine results in a reduction in processivity, indicated by the lack of shorter RNA products generated despite greater depletion of the intact 20-nt substrate compared to the wild-type reaction at the same enzyme concentration. (**b**) BLI sensograms showing the association and dissociation kinetics of the mutants. Titrated enzyme is in excess of the loaded dsRNA (same substrate as in Fig. 6). Best-fit curves to the single-site binding model are shown as dotted lines (*R*^2^ of 0.8289 and 0.958 for K9A and K139A, respectively). Due to K9A and K139A’s poor fitting of the data to the binding model, the kinetic parameters could not be determined reliably. Nonetheless, the diminished wavelength shifts of the mutants compared to the WT (Fig. 6a) suggest weaker binding.

We performed a structural comparison between nsp14-ExoN/10 and NP-ExoN with dsRNA in the active site of both enzymes to understand critical RNA-protein contacts that may be responsible for their difference in dsRNA binding affinity and kinetics (Fig. 7a and b) and investigated whether mutating residues involved in these contacts would result in a loss of processivity. The specifics of the polar contact comparison are elaborated on further in the discussion, along with buried surface area and electrostatic comparisons (Fig. 7c through e), but we chose to focus on nsp14-ExoN lysine residues involved in positioning RNA within the active site. To examine the role of these residues in the processivity of nsp14-ExoN/10, we mutated K9 and K139 to alanine and tested these mutants in our biochemical assays. The cryo-EM structure showed that K9 interacts with an RNA backbone phosphate group in the template strand directly across from the scissile phosphate (38) (Fig. 7b). K139 is located farther down along a basic patch toward the direction of the 5′ overhang of the template strand (Fig. 7b and e) and potentially involved in positioning RNA based on molecular modeling (42). The activities of wild-type nsp14-ExoN/10 and its lysine point mutants, K9A and K139A, were tested on the poly-U/A RNA in the single-hit condition. As described above for the comparison between the SARS-CoV-2 and LASV enzymes, RNA was held constant and in excess against an enzyme titration (Fig. 8a). Wild-type nsp14-ExoN/10 showed the characteristic processive behavior as shown above, generating a ladder pattern of heterogeneous degradation products down to mostly 5-mer and a faint 4-mer product (Fig. 8a, left). By contrast, the RNA degradation by K9A was more incremental and distributive (Fig. 8a, middle). For instance, comparing the reactions with a 9 nM enzyme, the wild-type left a greater amount of uncleaved 20-mer than K9A; however, the wild-type generated a 5-mer product, whereas the shortest product generated by K9A was a 9-mer. These data highlight a loss in processivity for K9A. While K139A showed a less dramatic effect, a tighter product distribution and less extensive RNA degradation compared to the wild- type, despite depletion of the intact 20-mer at a lower enzyme concentration, suggest a loss of processivity for K139A, too (Fig. 8a, right).

Finally, to corroborate the idea that dsRNA binding affinity and kinetics underlie the processivity of nsp14-ExoN/10, we used BLI to examine the binding of these lysine mutants (Fig. 8b). Unlike wild-type nsp14-ExoN/10, for which we were able to reliably determine the *K*_D_ and relevant kinetic parameters (Fig. 6a), the lysine mutants generated lower response signals, and the fit of the kinetic data to the single-site binding model was too poor to calculate meaningful rate constants. Nonetheless, the data suggested that mutating these lysine residues results in poorer binding, based on much reduced magnitudes of wavelength shift compared to wild-type. Compared to wild-type, which generated a ~0.7 nm shift at 1.25 μM analyte concentration, the same concentration of K9A generated only a ~0.1 nm shift, with more rapid dissociation (Fig. 8b, top). K139A generated a better signal of ~0.35 nm with a 1.25 μM analyte, but again with a more rapid dissociation compared to wild-type (Fig. 8b, bottom). Taken together, these results suggest that higher dsRNA binding affinity is important for higher processivity.

## DISCUSSION

SARS-CoV-2 nsp14-ExoN and LASV NP-ExoN share a common protein fold and specifically degrade dsRNA. Structural studies of the enzyme-dsRNA complexes showed that the conserved DEDDh active site of the two enzymes engages the scissile RNA strand of dsRNA substrates in similar fashions (37, 38) (Fig. 7a and b). However, sequence alignment shows that the RNA-interacting residues of these enzymes are distinct (Fig. S1). Eight polar contacts to the sugar-phosphate backbone of the scissile strand are observed in the nsp14/10 cryo-EM structure (38), while the NP-ExoN crystal structure shows six polar sugar-phosphate backbone contacts (37). Most of these scissile RNA-protein interactions for both nsp14/10 and NP-ExoN occur in the three 3′-terminal nucleotides. The difference in contacts is concentrated on the terminal cytosine of the scissile strand, where the polar atoms from the surrounding nsp14/10 residues interact twice as much as those from NP-ExoN. Both enzymes also make polar contacts with the non-scissile strand of dsRNA; nsp14/10 makes five hydrogen bonds, whereas NP-ExoN makes six (Fig. 7a and b). To investigate whether these RNA-contacting residues play a key role in the enzyme’s ability to processively degrade dsRNA, we tested a mutation of K9 of nsp14-ExoN/10 to an alanine in a single-hit RNA degradation gel assay and BLI experiments. Our biochemical data support that there is a correlation between dsRNA binding affinity and processivity (Fig. 8a and b). The nsp14-ExoN/10-dsRNA interface has a greater buried surface area (1,756 Å^2^) compared to the NP-ExoN-dsRNA interface (1,240 Å^2^) (Fig. 7c and d). In addition, the RNA-binding residues of nsp14/10 are flanked by a broader positively charged surface involving K139, whose mutation compromises the exonuclease activity (42) and may therefore contribute to the enzyme-RNA binding process. We therefore tested K139A in our biochemical studies and showed that this residue also plays a role in the nsp14-ExoN/10’s ability to processively degrade dsRNA, though not as critical as K9 (Fig. 8a and b). These structural differences in RNA contacts, buried surface area, and electrostatics may account for the higher dsRNA binding affinity and faster association/dissociation of nsp14/10, which would help the enzyme remain associated with the dsRNA substrate during multiple catalytic cycles.

The processive dsRNA degradation activity of nsp14/10 seems counterintuitive, given its well-established role in proofreading (11–13, 16), where the removal of a misincorporated nucleotide might best be achieved by a non-processive action of the exonuclease activity. However, a more processive action of the enzyme may benefit efficient degradation of viral pathogen-associated molecular pattern (PAMP) RNAs to suppress the host’s interferon response (20, 21). Retinoic acid-inducible gene I (RIG-I)-like receptors (RLRs) are cytosolic proteins that sense non-self RNAs such as PAMPs and stimulate the host type I interferon (IFN) response (50, 51). One PAMP that RIG-I specifically associates with is dsRNA with an exposed 5′ triphosphate. SARS-CoV-2 genomic RNA contains a 5′ cap (52), which aids in evading the immune response. Additionally, SARS-CoV-2 N protein antagonizes the RIG-I signaling pathway by physically binding RIG-I (53–55). Arenaviruses snatch the cap structure of host mRNAs to initiate viral transcription, and LASV NP’s N-terminal domain is responsible for binding and protecting the 5′ cap m7GpppN (24), evading key sensory interaction to induce the RIG-I signaling pathway.

While SARS-CoV-2 and LASV are well equipped to evade RIG-I sensing, they may lack in evasion of MDA5, another member of the RLR family. SARS-CoV-2 N protein inhibits RIG-I, but not MDA5, upon transfection (53). Nsp14/10 may aid in suppressing the MDA5-mediated host immune response where the N protein fails. Previous studies have shown that the Z proteins of pathogenic Arenaviridae inhibit both RIG-I and MDA5 (56). Lymphocytic choriomeningitis virus NP also physically associates with both RIG-I and MDA5, which suggests that these protein-protein interactions inhibit the type-I interferon response (57). Therefore, nsp14/10 may aid in MDA5 evasion, whereas LASV may already have protective measures against MDA5 detection.

It is also possible that nsp14/10 is responsible for degrading the negative-sense viral RNA strand in replication intermediates, where a highly processive ExoN activity may be required in replicating the large coronavirus genome. The ExoN activity of nsp14 was shown to be essential for SARS-CoV-2 replication (58, 59), and a loss of nsp14-ExoN activity impairs the replication, proofreading, fitness, and pathogenesis of SARS-CoV-2 (60), which may reflect its pleiotropic functions not limited to proofreading. Similarly, while the role of LASV NP in degrading immunostimulatory PAMP RNAs is well recognized, the viral ExoN activity has additional critical roles in supporting viral RNA replication (34), and the activity of NP-ExoN has likely been optimized to support its pleiotropic functions. The lack of enzyme processivity could perhaps be important in protecting the panhandle structure formed between the 5′ and 3′ termini of the viral genome, given that NP-ExoN resides in the C-terminus of the viral NP integral to the viral RNA encapsidation. Another possible explanation for NP-ExoN’s less processive activity is that other proteins involved in the LASV life cycle are necessary to aid in dsRNA clearance. During JUNV infection, both LASV NP and the LASV L protein are required to clear dsRNA production and inhibit the IFN response (61). As NP associates with L in order to form the RNP core for RNA replication and transcription (62–65), it could be that NP requires L interaction for more efficient excision of nucleic acid.

## MATERIALS AND METHODS

### Enzyme preparation

SARS-CoV-2 nsp14 and its N-terminal ExoN domain, nsp14 (1–289), were coexpressed with nsp10 in *Escherichia coli* and purified as reported previously (42). LASV NP containing only the C-terminal ExoN domain (NP-ExoN) was expressed with a C-terminal 6×His tag and purified similarly to full-length SARS-CoV-2 nsp14/10. Full-length NP (UniProt P13699) was purified similarly to NP-ExoN, with the exception of an additional heparin purification step after nickel affinity chromatography. The purified proteins were snap frozen in small-volume aliquots and stored at −80°C.

### Oligonucleotide preparation

RNA oligonucleotides (Integrated DNA Technologies) were dissolved in nuclease-free water, supplemented with HEPES-NaOH buffer (pH 7.5) and potassium acetate at the final concentration of 0.47 and 1.55 mM, respectively. Table 1 lists all oligonucleotide sequences and modifications. To prepare dsRNA assay stocks for the real-time fluorescence-based assay in enzyme titration experiments, the top (LS2U_RNA20_5′Cy3) and bottom (LS15A_IowaBlackRQ) strands were mixed, such that the final concentrations were 1.05 and 3.0 µM, respectively. dsRNA assay stocks for the same plate reader assay in RNA titration experiments were prepared in the same molar ratio and scaled up for a higher RNA concentration titration scheme.

### Real-time fluorescence-based assay

In the dsRNA titration experiments, enzyme and RNA were prepared in separate 40 µL reaction halves. Freshly thawed enzymes in storage buffer containing 20 mM Tris-HCl, pH 7.4, 500 mM NaCl, and 5 mM β-mercaptoethanol were centrifuged at 18,000 × *g* for 10 min at 4°C and then diluted in 1× reaction buffer containing 40 mM Tris-HCl, pH 8.0, 50 mM NaCl, 1.0 mM dithiothreitol (DTT), and 1.0 mM MgCl_2_ or MnCl_2_ such that the final concentration was 6 nM nsp14/10 and 86 nM NP-ExoN in the 80 µL total reaction mixture. The final enzyme concentrations for nsp14-ExoN/10 and NP were 24 and 86 nM, respectively. Five percent glycerol was included in a buffer containing 1 mM MgCl_2_ to supplement the nsp14/10 enzyme concentration. Before RNA was titrated, a mastermix of RNA diluted in the 1× buffer was heated for 1 min at 95°C and then slowly cooled to ensure complete annealing. RNA was titrated in 1× buffer in a 7-point serial dilution, where the highest condition tested was 500 nM and 1.3 µM LS2U final for the SARS-CoV-2 and LASV experiments, respectively. The RNA reaction halves were deposited by a multichannel pipettor into the black 96-well half-area plate (Corning 3993). After tapping the plate diagonally to remove air bubbles in the sample, the enzyme halves were mixed thoroughly into the same wells to start the reaction. Fluorescence scans were carried out using the TECAN Spark 10 M plate reader at 26°C using an excitation wavelength and bandwidth of 520 and 20 nm, respectively. The emission wavelength was set to 562 nm with a bandwidth of 20 nm. Instrument gain was set to 145, and the Z-position of the emitter was set to 17,732 µm. Total scan time was set to 22 and 42 min for the SARS-CoV-2 and LASV experiments, respectively, with 8 s scan intervals. Enzyme titration experiments followed a similar protocol, except that the enzymes were titrated in a 7-point twofold serial dilution, such that the highest final concentrations tested were 1.5 and 35.7 nM for nsp14/10 and NP-ExoN, respectively. The highest concentrations tested in these experiments for nsp14-ExoN/10 and NP were 10 and 35.7 nM, respectively. RNA concentration was held constant at final concentrations of 52.5 nM LS2U and 150 nM LS15A. The slopes of the kinetic traces were quantified using ICEKAT (66). The *K*_M_ values were determined by fitting data to the Michaelis-Menten model using GraphPad Prism.

### Time-resolved product distribution gel-based assays

6-FAM-labeled RNA oligonucleotide (LS2U_RNA20_5′FAM, Table 1) was annealed to a longer RNA oligonucleotide (LS15A_IowaBlackFQ, Table 1) to generate a dsRNA substrate with 20 duplexed bases with a 20-base 5′ overhang. This fluorescent-labeled RNA substrate was added at a final concentration of 52.5 nM to either nsp14/10 at 3.6 nM, nsp14-ExoN/10 at 13.5 nM, or both NP and NP-ExoN at 42 nM. The final reaction condition contained 40 mM Tris-HCl, pH 8.0, 52 mM NaCl, 1.0 mM DTT, 0.01% Tween 20, 2% glycerol, and 0.9 mM MgCl_2_ or MnCl_2_ for SARS-CoV-2 and LASV enzymes, respectively. Minute-by-minute time points were measured by stopping the reactions through the addition of formamide (eight parts into one part reaction) and loaded onto a 15% polyacrylamide TBE-urea gel, then run in TBE buffer at 300 V for 30 min. The gel was scanned using a Typhoon FLA 9500 imager to visualize the RNA products.

### Single-hit and spiked endpoint gel-based assay

6-FAM-labeled RNA oligonucleotide (U20_RNA_5′FAM, Table 1) was annealed to a longer RNA oligonucleotide (A30_RNA, Table 1) to generate a dsRNA substrate. This fluorescently labeled RNA was added at a final concentration of 500 nM to either nsp14/10, nsp14-ExoN/10, NP, or NP-ExoN serially diluted to the stated concentrations. The final reaction condition contained 40 mM Tris-HCl, pH 8.0, 52 mM NaCl, 1.0 mM DTT, 0.01% Tween 20, 2% glycerol, and 0.9 mM MgCl_2_ or MnCl_2_ for SARS-CoV-2 and LASV enzymes, respectively. Endpoint gel-based assays with spiked enzyme concentrations were performed in a similar manner. After 15 min, the reactions were stopped by the addition of formamide (eight parts into one part reaction) and loaded onto a 15% polyacrylamide TBE-urea gel, then run in TBE buffer at 300 V for 30 min. The gel was scanned using a Typhoon FLA 9500 imager to visualize the RNA products.

### Biolayer interferometry

5′-Biotinylated LS2U RNA (Table 1) was diluted to a concentration of 50 nM in assay buffer containing 20 mM HEPES-NaOH, pH 8.0, 165 mM, 1.0 mM DTT, 5% glycerol, and 0.01% Tween 20. LS15A_RNA-ddC (Table 1) was diluted to a concentration of 150 nM in the same buffer. Nsp14/10 was serially diluted to the concentration range from 750 to 93.8 nM, with the final buffer condition adjusted to the same as above. Nsp14-ExoN/10 was serially diluted to the concentration range of 1,250–165 nM in the assay buffer. NP was serially diluted in the same assay buffer to the concentration range of 1,400–184 nM. NP-ExoN was similarly prepared in the range from 25 to 3.13 µM. The concentration range for each enzyme was chosen based on a pilot experiment to achieve signal saturation in the raw kinetic traces. To remove background noise during data analysis, an additional reference sample, which contained only the assay buffer, was added to each dilution series. All experiments were performed on an Octet RED384 using SAX Biosensors. Biosensor tips were pre-hydrated in the assay buffer for 10 min before collecting a background reading in a 45 μL buffer for 200 s. Biosensor tips were then dipped into 45 μL of 50 nM biotinylated LS2U for 150 s to load the top strand, dipped into buffer-only conditions for 80 s to wash off excess substrate, then dipped into 150 nM LS15AddC to anneal the bottom strand to LS2U bound to the biosensors. After loading the dsRNA, the biosensor tips were dipped into 45 μL of buffer to remove unbound bottom strand and to measure the background signal for 80 s. The protein association rate was measured by dipping the dsRNA-loaded biosensor tips into 45 μL of their respective dilution of nsp14/10, nsp14-ExoN/10, and NP-ExoN for 300 s. The association phase for NP involved dipping the sensors into the sample for 600 s to fully capture the signal plateau. For all samples, the biosensor tips were then dipped into 45 μL of buffer for 600 s to measure the dissociation rate. All data analysis was performed using the Octet BLI Data Analysis HT 11.1 software.

## Data Availability

All data are available from the authors upon request.

## SUPPLEMENTAL MATERIAL

Supplemental figures jvi.00798-26-s0001.pdf Fig. S1 to S9.

ASM does not own the copyrights to Supplemental Material that may be linked to, or accessed through, an article. The authors have granted ASM a non-exclusive, world-wide license to publish the Supplemental Material files. Please contact the corresponding author directly for reuse.
