# Supplementary material for: Distinct modes of RNA degradation by the structurally related coronavirus and arenavirus exoribonucleases
Source: J Virol. 2026 Jul 23;100(8):e00798-26. doi: 10.1128/jvi.00798-26 (PMC13483370; doi:10.1128/jvi.00798-26)
Supplement: Supplementary file 1 [file jvi.00798-26-s0001.pdf]

Supplementary information

Distinct modes of RNA degradation by the structurally related coronavirus and arenavirus exoribonucleases

Hernandez *et al.*

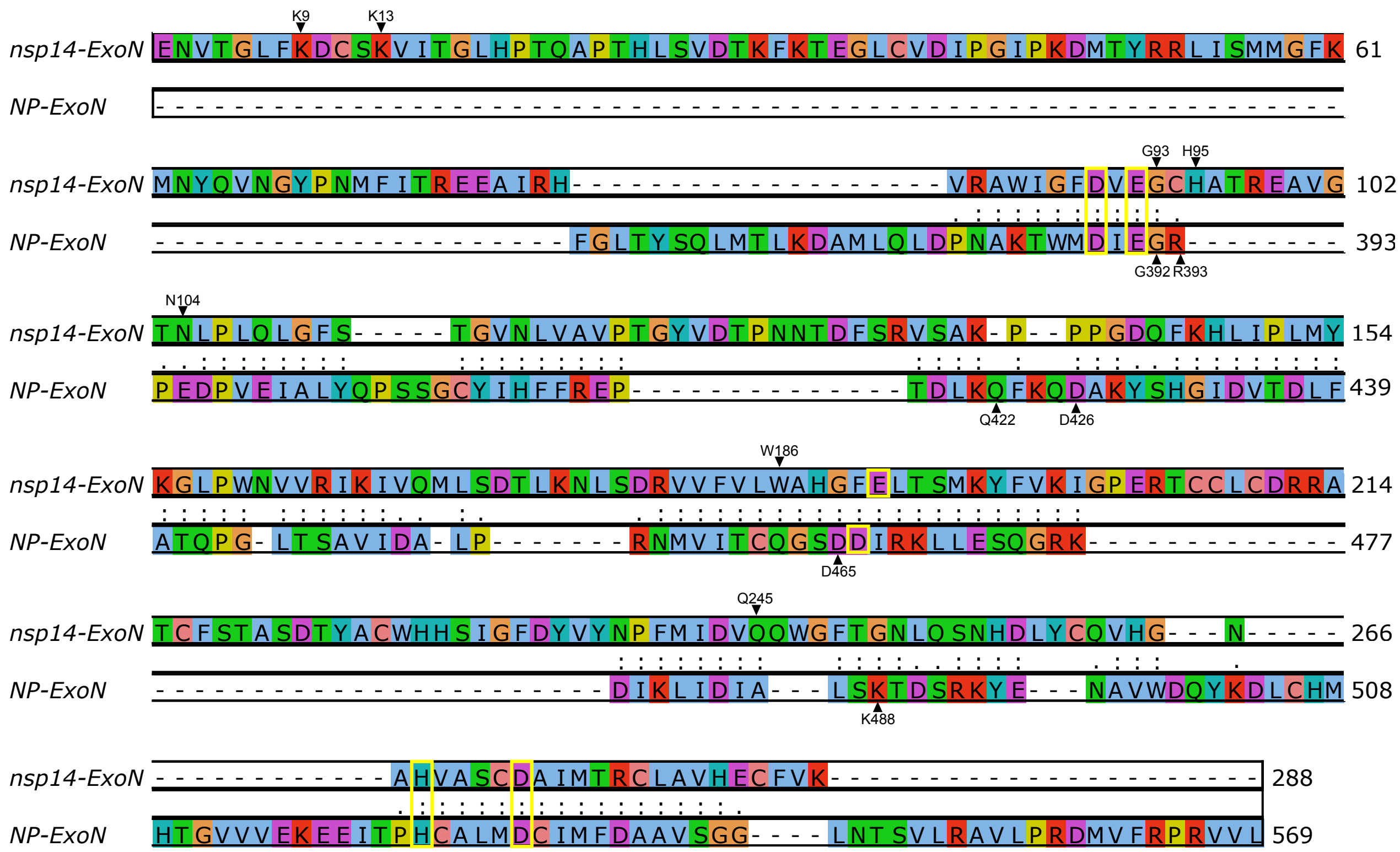

Supplementary Figure 1. Structure-based amino acid sequence alignment between the exonuclease domains of SARS-CoV-2 nsp14 and LASV NP. The sequence alignment was performed using TM-align (1). The DEDDh motif is highlighted by yellow boxes. RNA-contacting residues are indicated. Residues with a colon indicate structural alignment with a distance of less than 5.0 Å, and residues marked with a period denote other aligned residues.

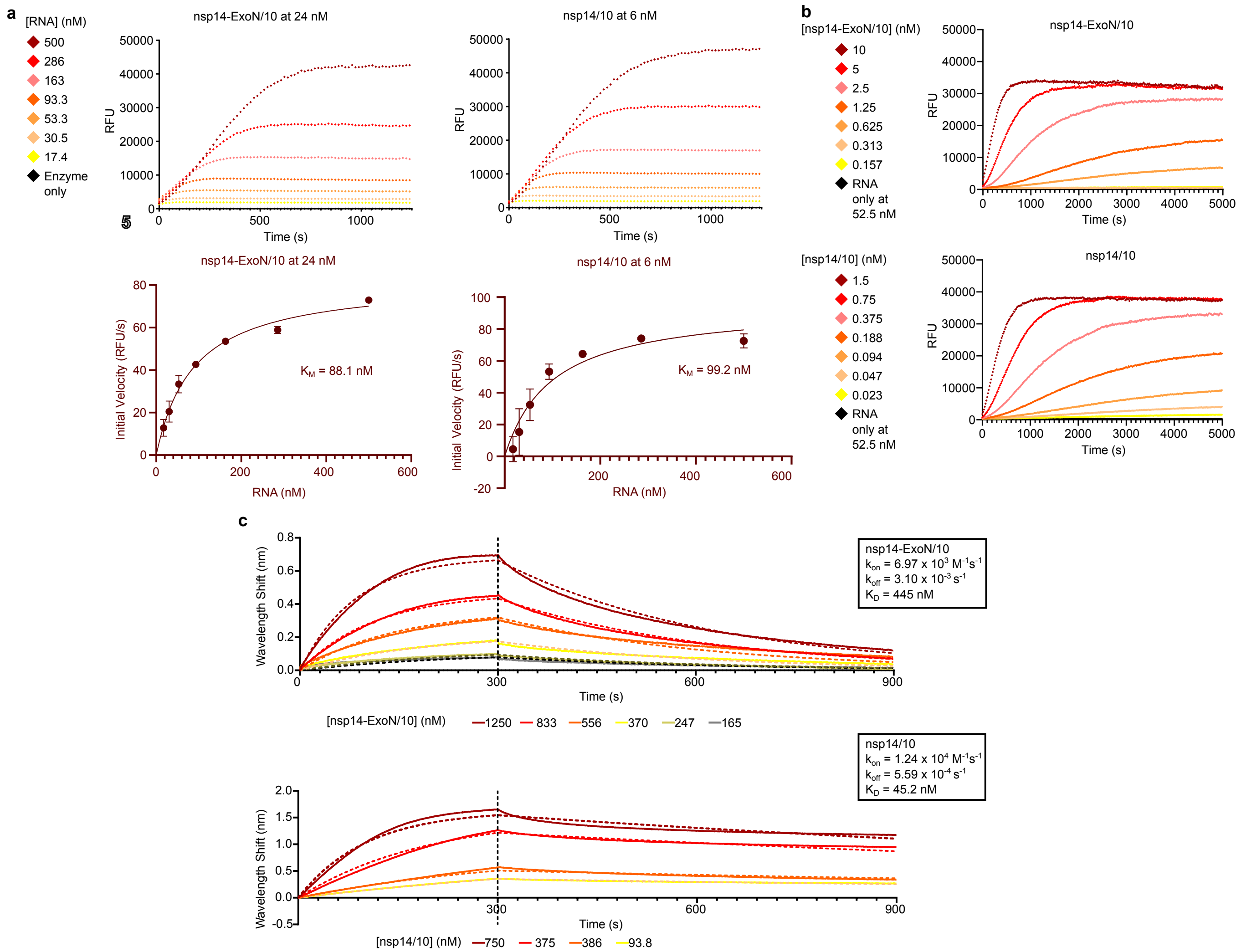

Supplementary Figure 2. Comparison of the exonuclease domain and full-length versions of SARS-CoV-2 nsp14 with nsp10 in various biochemical assays. a, Real-time fluorescence traces (top row), where enzyme concentration is fixed at 24 and 6 nM for nsp14-ExoN/10 and nsp14/10, respectively. Substrate is titrated in a dilution series beginning at 500 nM (LS2U_RNA20_5'Cy3 annealed to LS15A_IowaBlack®RQ; Table 1). The corresponding curves (bottom row) show fitting of raw data to the Michaelis-Menten model (R^2 of 0.973 and 0.888 for nsp14-ExoN/10 and nsp14/10, respectively). The K_M values were determined to be 88.1 nM with 95% CI [71.9, 108] and 99.2 nM with 95% CI [64.3, 154] for nsp14-ExoN/10 and nsp14/10, respectively. The same data are in Fig. 2a, b. **b**, Enzyme titration of nsp14-ExoN/10 and nsp14/10 in the real-time fluorescence-based assay with the 20-mer substrate. Substrate is fixed at 52.5 nM and enzyme is titrated in a two-fold dilution series beginning at 10 and 1.5 nM for nsp14-ExoN/10 and nsp14/10, respectively. **c**, BLI sensograms showing the association and dissociation kinetics of nsp14-ExoN/10 and nsp14/10 to immobilized dsRNA (LS2U_RNA20_5'Biotin and LS15A_RNA-ddC; Table 1), where titrated enzyme is in excess of the loaded dsRNA. Best-fit curves to the single-site binding model are shown as dotted lines (R^2 of 0.992 for both enzymes). The same data are in Fig. 6a, c.

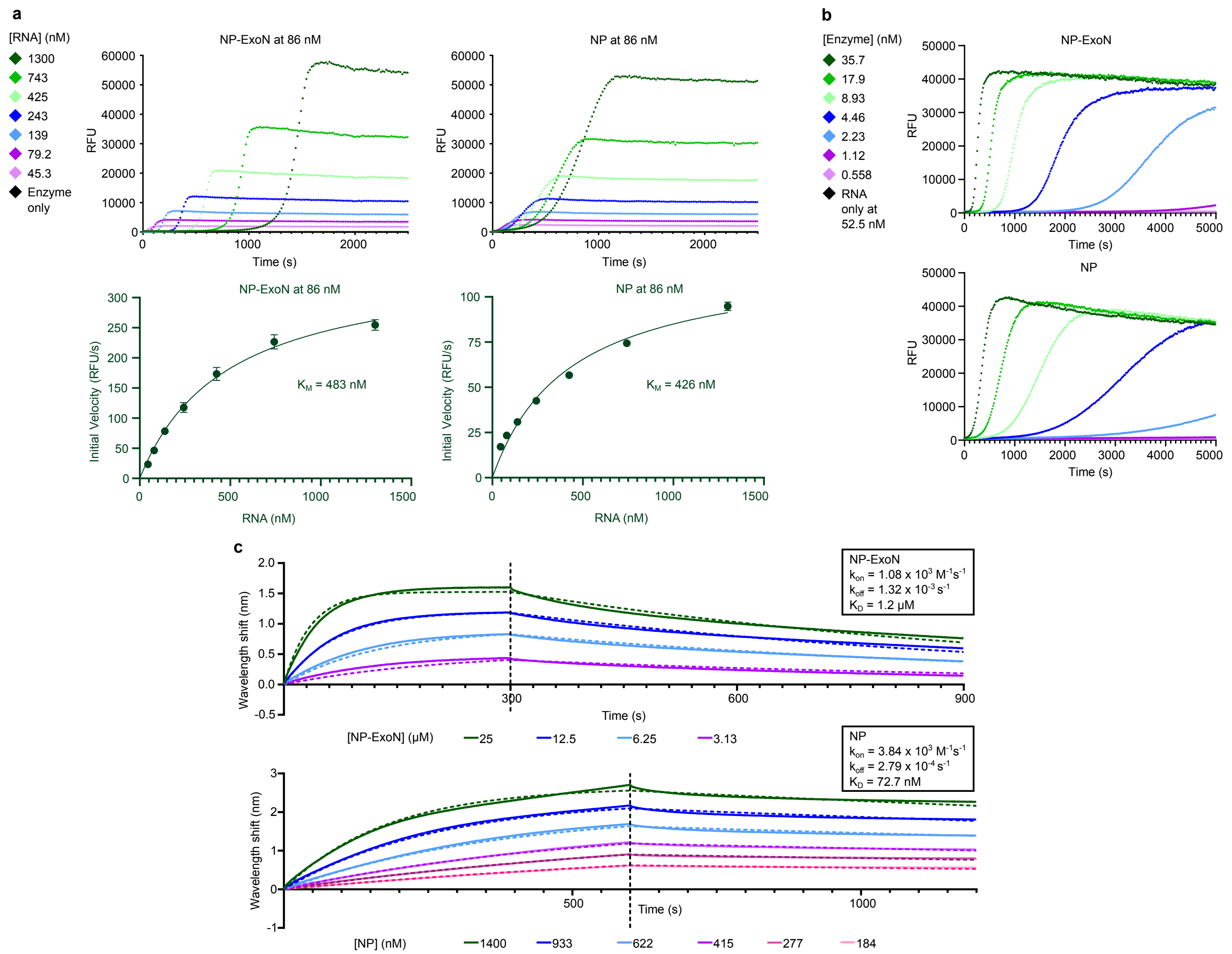

Supplementary Figure 3. Comparison of the exonuclease domain and full-length versions of Lassa virus nucleoprotein in various biochemical assays. a, Real-time fluorescence traces (top row), where enzyme concentration is fixed at 86 nM and dsRNA is titrated in a dilution series beginning at 1300 nM (LS2U_RNA20_5'Cy3 annealed to LS15A_IowaBlack®RQ; Table 1). The corresponding curves (bottom row) show fitting of raw data to the Michaelis-Menten model (R^2 = 0.989 and 0.981 for NP-ExoN and NP, respectively). The K_M values were determined to be 483 nM with 95% CI [411, 570] and 426 nM with 95% CI [346, 527] for NP-ExoN and NP, respectively. The same data are in Fig. 2c, d. **b**, Enzyme titration of NP-ExoN and NP in the real-time fluorescence-based assay with the 20-mer substrate, where dsRNA is fixed at 52.5 nM and enzyme is titrated in a two-fold dilution series beginning at 35.7 nM. **c**, BLI sensograms showing the association and dissociation kinetics of NP-ExoN and NP to immobilized dsRNA (LS2U_RNA20_5'Biotin and LS15A_RNA-ddC; Table 1), where titrated enzyme is in excess of the loaded dsRNA. Best-fit curves to the single-site binding model are shown as dotted lines (R^2 of 0.990 and 0.998 for NP-ExoN and NP, respectively). The same data are in Fig. 6b, d.

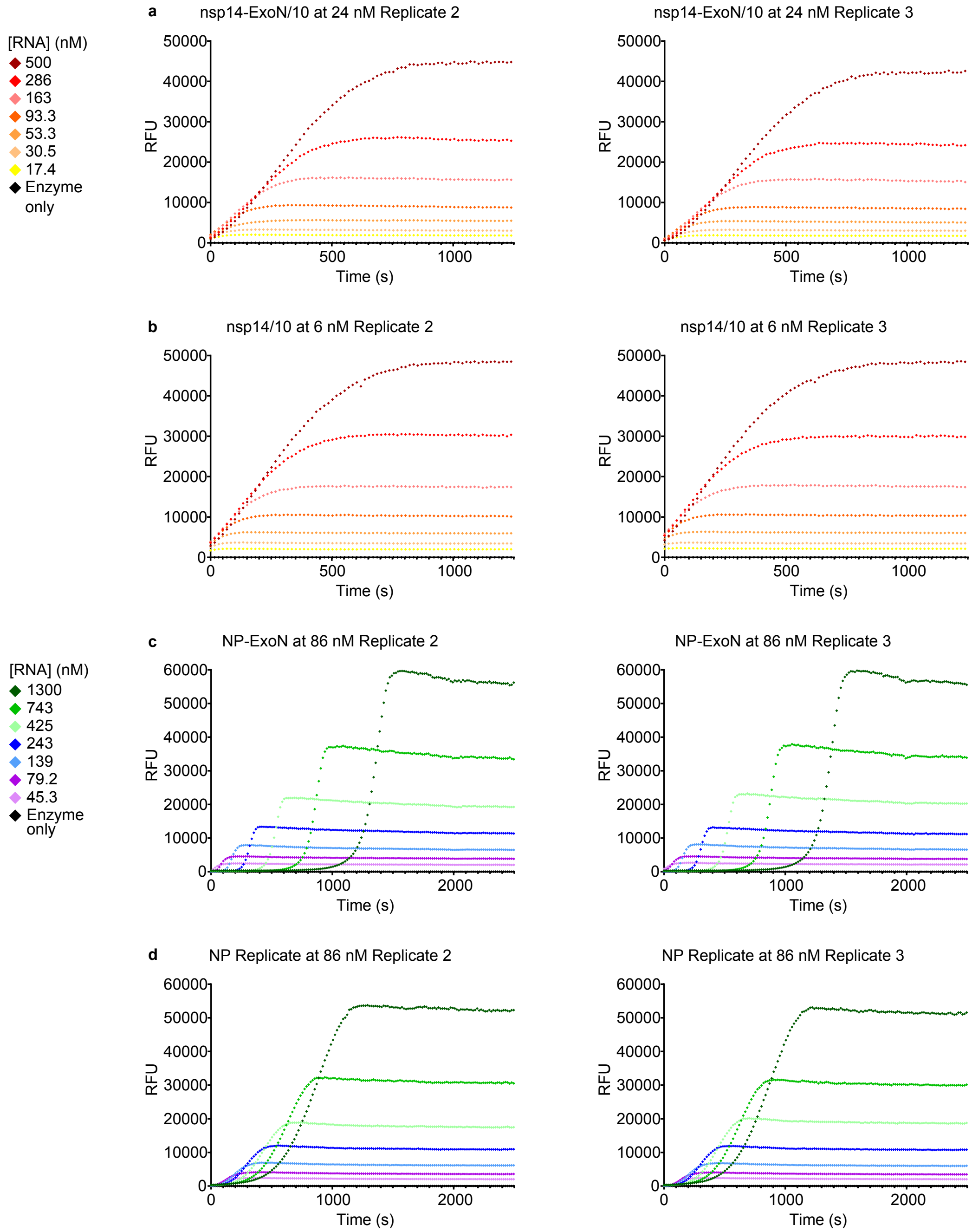

Supplementary Figure 4. Replicates of dsRNA titration experiments. Each graph in the dsRNA titration experiments shown in Fig. 2 represents the results of triplicate experiments of kinetic traces, as shown above. Error bars in the Michaelis-Menten curves represent the standard deviation amongst the triplicate data.

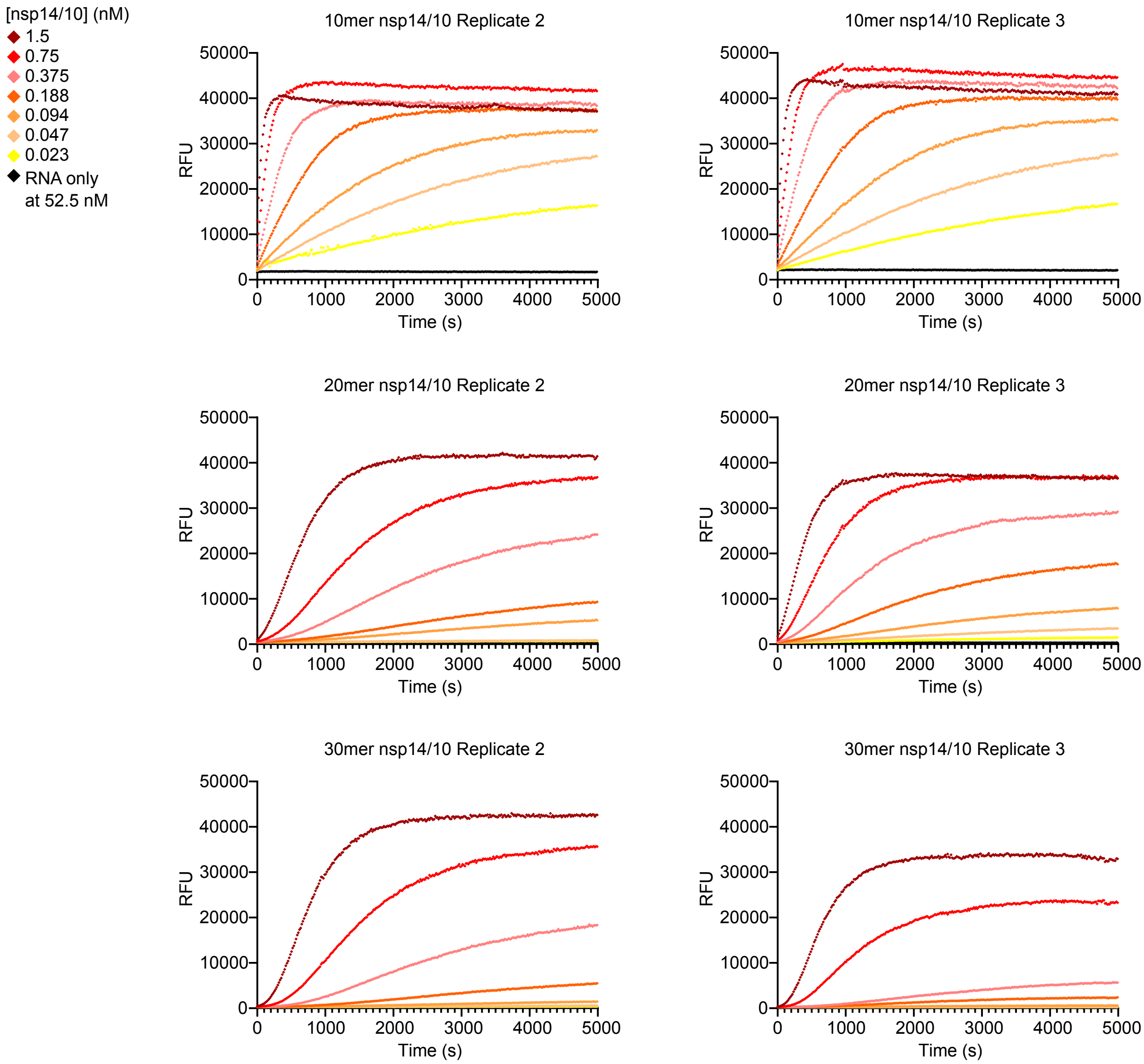

Supplementary Figure 5. Replicates of nsp14/10 titration experiments against varying top strand lengths of dsRNA substrates. Each nsp14/10 graph in Fig. 3a is representative of triplicate experiments, shown above. Error bars in the slope comparison analyses (Fig. 4) represent the standard deviation among the triplicate data.

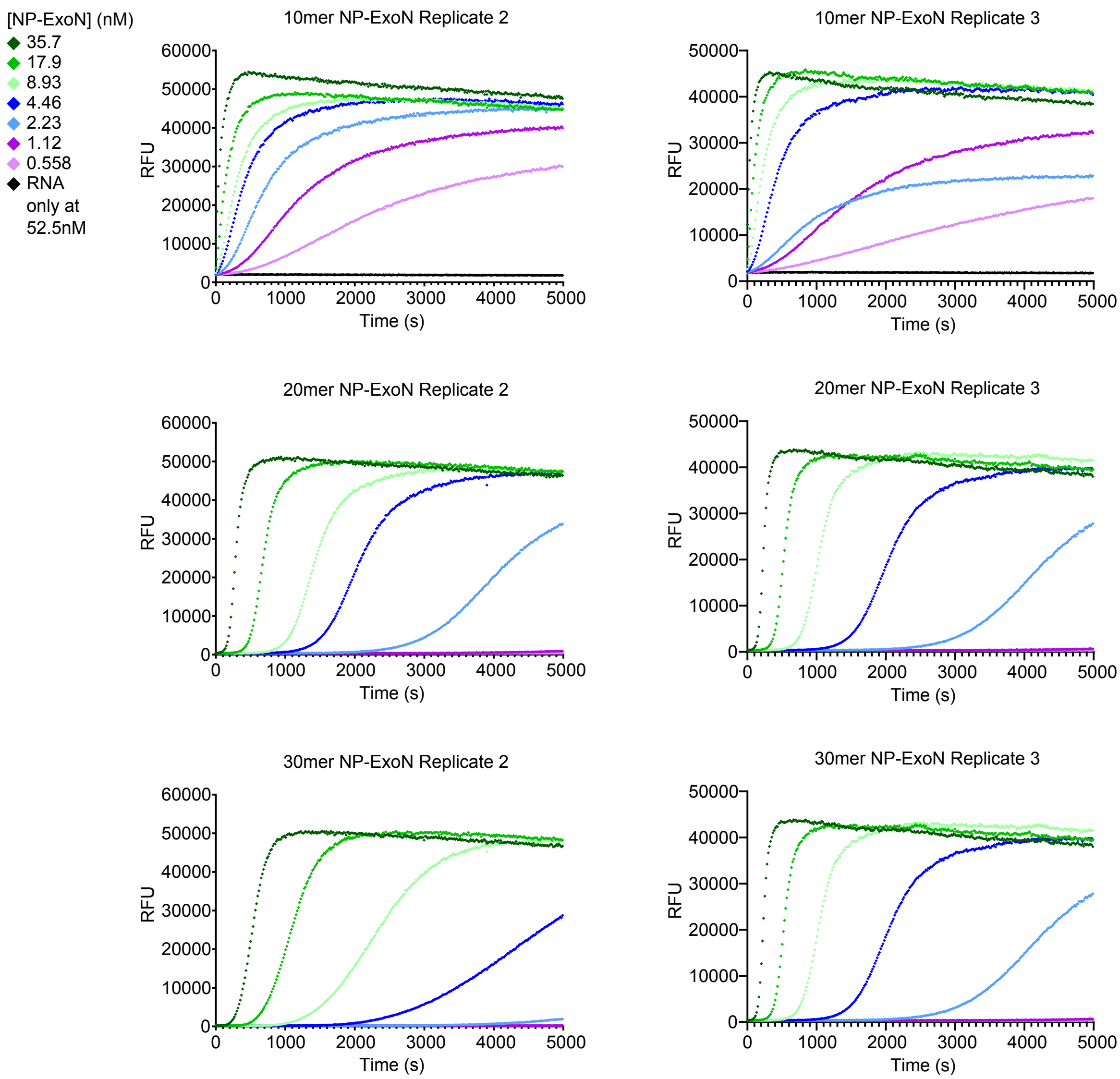

Supplementary Figure 6. Replicates of NP-ExoN titration experiments against varying top strand lengths of dsRNA substrates. Each NP-ExoN graph in Fig. 3b represents triplicate experiments, as shown above. Error bars in the slope comparison analyses (Fig. 4) represent the standard deviation among the triplicate data.

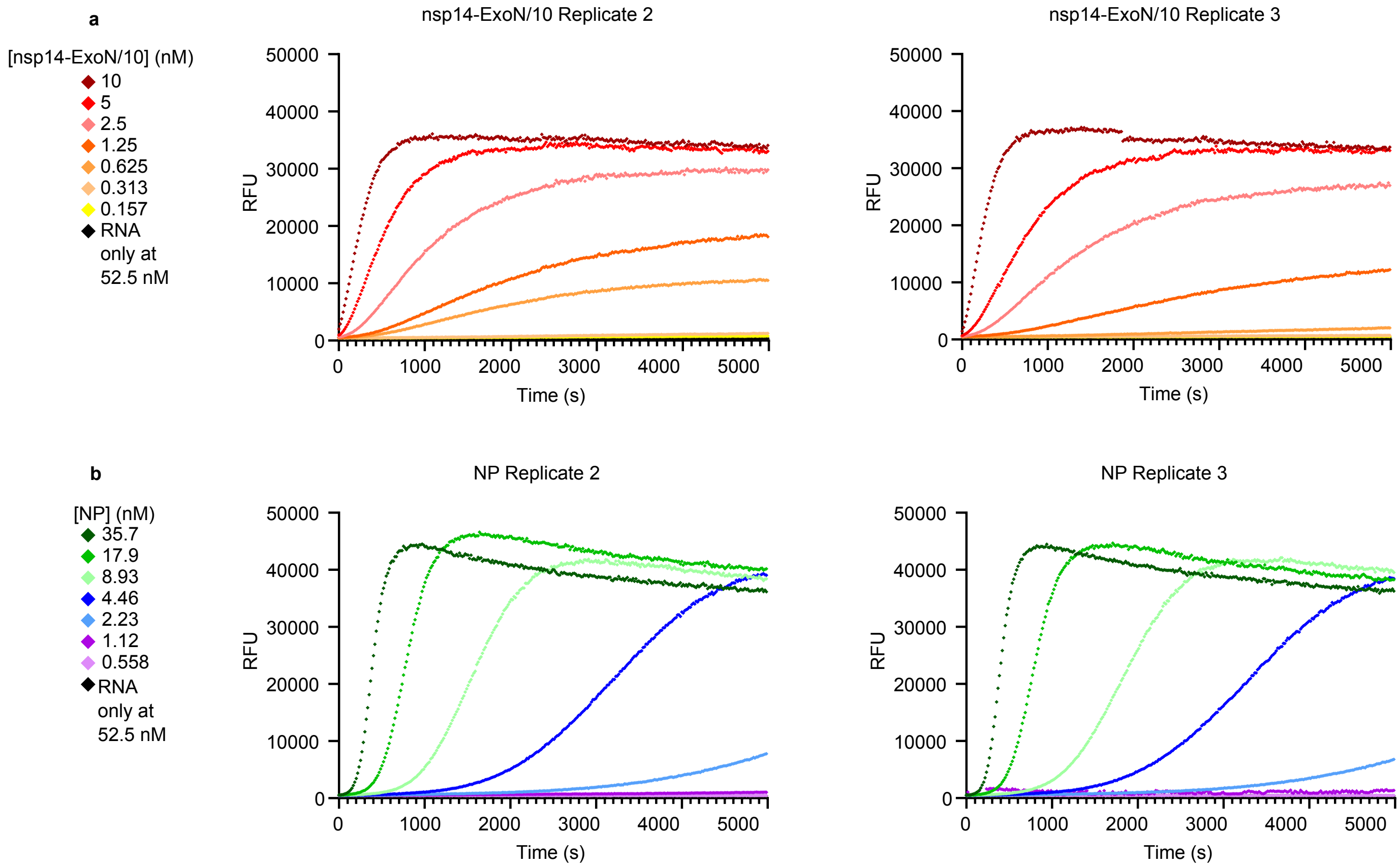

Supplementary Figure 7. Replicates of nsp14-ExoN/10 and NP titration experiments in the real-time kinetic assay with the 20-mer substrate. Each nsp14/ExoN (**a**) and NP **(b**) graph is a replicate of those in Supplementary Figs. 2b and 3b.

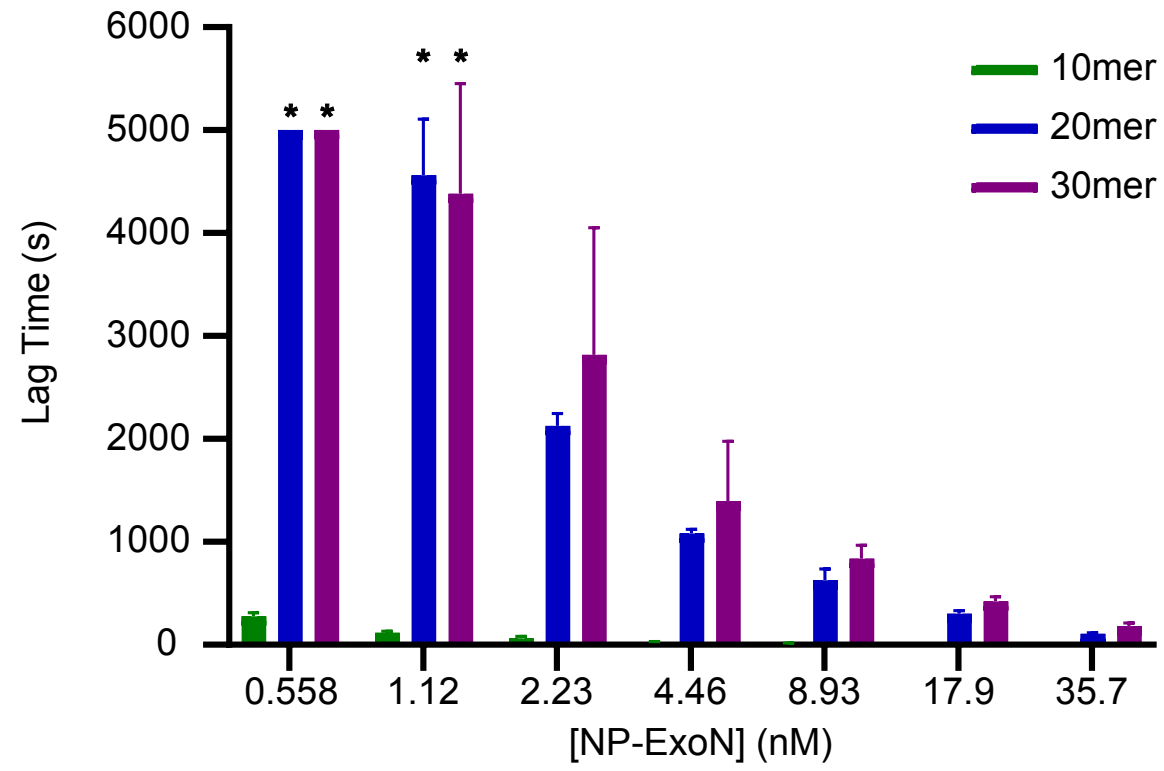

Supplementary Figure 8. NP-ExoN lag-time measurements for the fluorescence-based RNA degradation kinetic traces in Fig. 3. "Lag-time" was defined as the amount of time each reaction took to exceed 1% of the maximum fluorescence, corresponding to the fully degraded state of the same substrate. NP-ExoN concentrations marked with an asterisk indicate that 1% of the maximum fluorescence was never reached during the duration of the experiment for at least one of the replicates. Error bars represent the standard deviation among triplicate experiments.

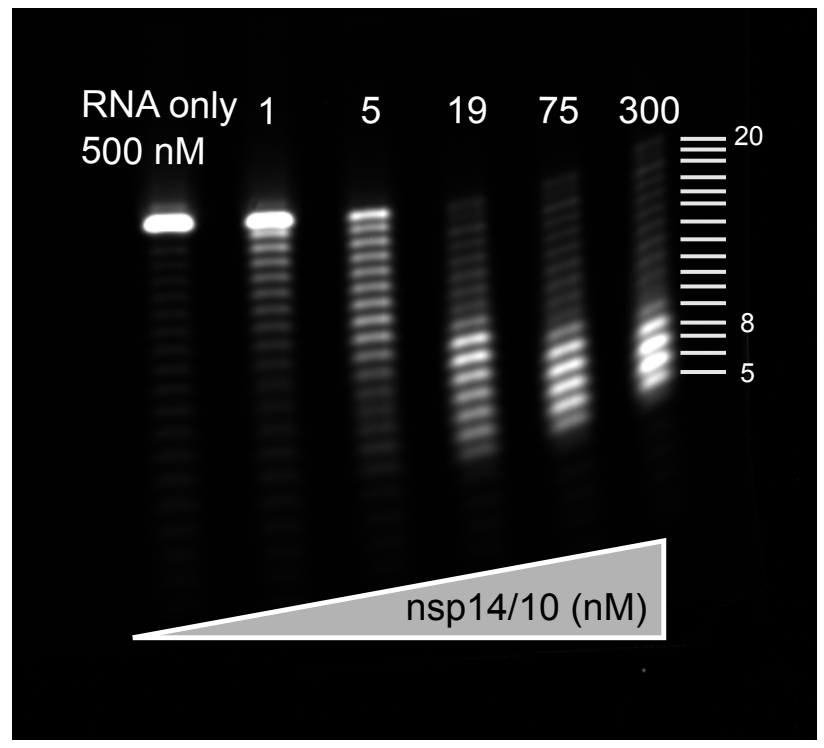

Supplementary Figure 9. Saturating nsp14/10 concentration does not fully degrade dsRNA (related to Fig. 5e-g). A pilot experiment to determine a suitable nsp14/10 concentration range against a fixed concentration of homopolymer dsRNA (U20_RNA_5'FAM annealed to A30_RNA; Table 1). RNA was fixed at 500 nM. Increasing nsp14/10 concentration up to 300 nM does not degrade the oligonucleotide to shorter than a 5-mer, showing the limit of ExoN-mediated degradation.

Supplementary references

1. Zhang Y, Skolnick J. 2005. TM-align: a protein structure alignment algorithm based on the TM-score. Nucleic Acids Res 33:2302-9.
